# Molecular and morphological characterisation of *Diplostomum phoxini* (Faust, 1918) with a revised classification and an updated nomenclature of the species-level lineages of *Diplostomum* (Digenea: Diplostomidae) sequenced worldwide

**DOI:** 10.1017/S0031182021001372

**Published:** 2021-11

**Authors:** Jessica Schwelm, Simona Georgieva, Daniel Grabner, Aneta Kostadinova, Bernd Sures

**Affiliations:** 1 Aquatic Ecology and Centre for Water and Environmental Research, University of Duisburg-Essen, Universitätsstraße 5, D-45141 Essen, Germany; 2 Institute of Biodiversity and Ecosystem Research, Bulgarian Academy of Sciences, 2 Gagarin Street, 1113 Sofia, Bulgaria; 3 Science Park, Cavanilles Institute of Biodiversity and Evolutionary Biology, University of Valencia, Valencia, Spain

**Keywords:** *Ampullaceana balthica*, *Diplostomum baeri* species complex, *Diplostomum phoxini*, Germany, Molecular phylogeny, *Phoxinus phoxinus*, River Ruhr

## Abstract

We characterised morphologically and molecularly *Diplostomum phoxini* (Faust, 1918) based on cercarial isolates from the snail *Ampullaceana balthica* (L.) (Gastropoda: Lymnaeidae) and metacercariae from the Eurasian minnow, *Phoxinus phoxinus* (L.) (Cypriniformes: Leuciscidae), and provided molecular evidence for the identification of the snail intermediate host. Phylogenetic analyses based on the cytochrome *c* oxidase subunit 1 (*cox*1) gene depicted 44 molecularly characterised species and genetically distinct lineages of *Diplostomum*, and resulted in: (i) a re-identification/re-classification of 98 isolates plus *D. baeri* sampled in North America; (ii) re-definition of the composition of the *D. baeri* species complex which now includes nine molecularly characterised species/lineages; (iii) re-definition of the composition of the *D. mergi* species complex which now includes seven molecularly characterised species/lineages; and (iv) an updated nomenclature for the molecularly characterised species-level lineages of *Diplostomum*.

## Graphical abstract

**Figure:**
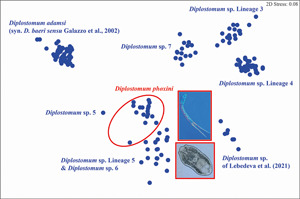

## Introduction

The application of molecular tools for characterisation and phylogenetic analyses has greatly advanced our understanding of the diversity, taxonomy, systematics and phylogeny of virtually all major groups of parasitic worms. Molecular data have become a ‘must-have’ characteristic not only in species discovery and delineation but also in large-scale biodiversity inventories, and ecological and evolutionary research. This is especially true for the trematode subclass Digenea, parasitic flatworms representing a remarkable example of the diversity of complex life-cycles among the Metazoa (Minelli and Fusco, 2010), which involve alternation of generations and a diversity of phenotypes in the sequential hosts in the life-cycle.

The digenean genus *Diplostomum* von Nordmann, 1832 (Diplostomidae) has received increased attention in recent years. Intensive sampling and molecular analyses predominantly of the larval stages of *Diplostomum* spp. from their intermediate hosts, freshwater lymnaeid snails and fishes, have resulted in delineation of more than 40 species/species-level lineages (Locke *et al*., 2010*a*, 2010*b*, 2015, 2020; Georgieva *et al*., 2013; Blasco-Costa *et al*., 2014; Faltýnková *et al*., 2014; Pérez-del-Olmo *et al*., 2014; Selbach *et al*., 2015; Kudlai *et al*., 2017; Soldánová *et al*., 2017; Gordy and Hanington, 2019; Hoogendoorn *et al*., 2020; Lebedeva *et al*., 2021). Complete mitochondrial genomes have been characterised for four species, *Diplostomum spathaceum* (Rudolphi, 1819) and *D. pseudospathaceum* Niewiadomska, 1984 (see Brabec *et al*., 2015), *D. ardeae* Dubois, 1969 (see Locke *et al*., 2020) and *Diplostomum baeri* Dubois, 1937 (see Landeryou *et al*., 2020).

However, the number of named molecularly characterised species remains low because of the difficulties in gathering adult worms from their definitive hosts (fish-eating birds) and the virtual lack of taxonomic expertise in identification of the larval stages. These include *D. spathaceum* (Rudolphi, 1819) (type-species), *D. ardeae* Dubois, 1969, *D. baeri sensu* Galazzo *et al*. (2002), *D. huronense* (La Rue, 1927), *D. indistinctum* (Guberlet, 1922), *D. lunaschiae* (Locke, Drago, Núñez, Rangel e Souza & Takemoto, 2020), *D. pseudospathaceum* Niewiadomska, 1984 and *D. parviventosum* Dubois, 1932. Phylogenetic analyses have depicted two species complexes among the prevailing unnamed species-level lineages. The *D. mergi* complex comprises one named species and three species-level lineages (*D. parviventosum*; *D. mergi* Lineages 2 and 3 of Georgieva *et al*. (2013); and *D. mergi* Lineage 4 of Selbach *et al*. (2015)), and the *D. baeri* complex *sensu* Blasco-Costa *et al*. (2014) comprises one named species and seven species-level lineages (*D. baeri sensu* Galazzo *et al*. (2002), *Diplostomum* sp. Lineages 3–5 of Blasco-Costa *et al*. (2014), *Diplostomum* sp. 2 of Moszczynska *et al*. (2009), and *Diplostomum* spp. 5–7 of Locke *et al*. (2010*a*)). Of these, seven lineages have been sequenced and morphologically characterised in Europe based on larval isolates (Blasco-Costa *et al*., 2014; Faltýnková *et al*., 2014; Selbach *et al*., 2015; Lebedeva *et al*., 2021).

In a study of larval digenean communities in the snail host *Ampullaceana balthica* (L.) (Gastropoda: Lymnaeidae) in the River Ruhr drainage, we collected a number of cercarial isolates which at first glance resembled morphologically the known cercariae of the *D. baeri* species complex. However, sequencing of the cytochrome *c* oxidase subunit 1 (*cox*1) mitochondrial gene indicated high similarity with two isolates of *Diplostomum phoxini* (Faust, 1919) collected in Norway (Soldánová *et al*., 2017). Therefore, we sampled the specific second intermediate host of this species, *Phoxinus phoxinus* (L.) (Cypriniformes: Leuciscidae), and sequenced the metacercariae recovered from the brain of the fish. Here, we provide molecular and morphological characterisation of *D. phoxini*, one of the few species of *Diplostomum* exhibiting strict host specificity to the second intermediate host (*P. phoxinus*), and molecular evidence for the identification of its first intermediate host (*A. balthica*). Phylogenetic analyses revealed changes in the composition of the *D. baeri* and *D. mergi* species complexes, and resulted in a re-classification of a large number of sequenced isolates. Finally, we compare the prevalence of *D. phoxini* and the molecularly characterised species/lineages of the *D. baeri* complex in the lentic and lotic aquatic habitats of Europe.

## Materials and methods

### Sample collection and examination

A total of 1599 *Ampullaceana balthica* (Gastropoda: Lymnaeidae; formerly often reported as *Radix balthica*) were collected and examined for trematode infections during spring (May), summer (June, July, August), autumn (September, October, November) and winter (December) in 2016 and 2017 and from May to September in 2019. Snails were collected at three sampling sites at the River Ruhr at Neheim (North Rhine-Westphalia, Germany): B0 (51°26′24.4″N, 7°58′35.8″E); B1 (51°26′25.9″N, 7°57′50.9″E) and B2 (51°26′53.2″N, 7°57′09.5″E). All snails were collected by hand and using a strainer from stones, driftwood and macrophytes, or picked directly from the sediment in shallow, slow-moving parts of the river. In the laboratory, snails were measured (shell width and height) and placed separately in containers with filtered river water under a light source to stimulate the emergence of cercariae. All containers were checked under light microscope for three consecutive days for the presence of cercariae in the water column. On the fourth day, all snails were dissected and examined for the presence of prepatent infections (sporocysts) (as described, e.g. in Selbach *et al*., 2015; Schwelm *et al*., 2018). Additionally, 15 specimens of the Eurasian minnow *Phoxinus phoxinus* (L.) were sampled *via* electrofishing at one site of the River Ruhr at Arnsberg (B4) (51°24′04.3″N, 8°04′01.1″E) in June 2019. In the laboratory, all fish were identified using Kottelat and Freyhof (2007), dissected and investigated for the presence of metacercariae in the brain.

Cercariae and metacercariae were fixed in molecular grade ethanol for DNA isolation and sequencing, and in 4% formaldehyde solution for morphological analyses (scanning electron microscopy, SEM). Cercariae studied by SEM were cleaned and subsequently post-fixed in 1% osmium tetroxide for 2 h, washed in 0.1 M phosphate buffer, dehydrated in an ethanol series, critical-point dried, sputter-coated with gold and examined and photographed with a scanning electron microscope (Hitachi 4100 FE Ltd., Tokio, Japan) at 20 kV at the Central Service for Experimental Research (SCSIE), University of Valencia, Spain. Foot tissue from snails was fixed in molecular grade ethanol for molecular identification.

### Morphological data

Trematode larval stages were identified live using light microscopy (Olympus BX51, Tokyo, Japan). Cercariae and metacercariae were identified to the species level based on the morphological descriptions of Arvy and Buttner (1954), Rees (1957) and Dönges (1969*a*, 1969*b*). Series of detailed light microscopy photographs of cercariae and metacercariae of *D. phoxini* were taken with a digital camera (Olympus UC30, Tokyo, Japan) attached to the light microscope and all visible features were recorded. Descriptions of the cercariae are based on examination of live material and digital photomicrographs from both, light microscopy and SEM. Measurements were taken with the program ImageJ 1.47v (available from https://imagej.nih.gov/ij/download.html) and are given in micrometres as the range followed by the mean in parentheses. The following abbreviations were used in the description of the cercaria: AOW, anterior organ width; BL, body length; FL, furca length; TSL, tail stem length; VSW, ventral sucker width.

### Molecular data

Total genomic DNA (gDNA) was isolated from ethanol-fixed snail tissue, pooled samples of 10–15 cercariae or single metacercariae by placing the samples in 200 *μ*L of a 5% suspension of deionised water and Chelex^®^, containing 0.1 mg mL^−1^ proteinase K, followed by incubation at 56°C for 3 h, boiling at 90°C for 8 min, and centrifugation at 14 000× *g* for 10 min. Polymerase chain reaction (PCR) amplification was carried out using 2× MyFi Mix (Meridian Bioscience, Cincinnati, USA), 8 pmol of each primer and *c*.50 ng of gDNA in a total volume of 20 *μ*L. Partial fragments of the mitochondrial cytochrome *c* oxidase subunit 1 (*cox*1) gene, the nuclear 28S rRNA gene and the complete ITS1-5.8S-ITS2 gene cluster (ITS2 only for *A*. *balthica*) were sequenced for the snail host and larval stages of *D. phoxini* using the primers and cycling conditions listed in Online Resource Table S1.

PCR amplicons were purified using QIAquick PCR purification kit (Qiagen Ltd, Hilden, Germany) following the manufacturer's instructions. PCR fragments were sequenced directly with ABI BigDye chemistry (ABI Perkin-Elmer, UK), alcohol-precipitated and run on an ABI Prism 3730XL DNA analyser using the primers listed in Online Resource Table S1.

Newly generated and published sequences were aligned with MAFFT v.7 (Kuraku *et al*., 2013; Katoh *et al*., 2019). The *cox*1 sequences were aligned with reference to the amino acid translation, using the echinoderm and flatworm mitochondrial code (translation table 9; Telford *et al*., 2000) for parasite isolates and the invertebrate mitochondrial code (translation table 5) for snail host isolates; the alignments contained no insertions or deletions.

Molecular identification/delimitation of the snail and parasite samples was achieved using neighbour-joining (NJ) analyses of Kimura 2-parameter distances for the *cox*1 alignments conducted with MEGA v.7 (Kumar *et al*., 2016); nodal support was estimated using 1000 bootstrap replicates. Genetic distances (uncorrected p-distance) were calculated with MEGA v.7. Non-metric multidimensional scaling (NMDS) plot was generated with Primer v.6 software (Anderson *et al*., 2008) to visualize the raw pairwise distances between species/lineages of the *D. baeri* species complex. Unique haplotypes were identified with DnaSP (Rozas *et al*., 2003) against the recently published sequences for *D. phoxini* by Lebedeva *et al*. (2021).

Species relationships within *Diplostomum* were assessed using Bayesian inference (BI) analysis of *cox*1 data. Prior to analysis, the best-fitting model of nucleotide substitution (HKY + Г + I) was estimated based on the Bayesian information criterion (BIC) using jModelTest v. 2.1.4 (Darriba *et al*., 2012). BI analysis was carried out with MrBayes v. 3.2.7 (Ronquist *et al*., 2012) on the CIPRES Science Gateway v.3.3 (Miller *et al*., 2010) using Markov chain Monte Carlo searches on two simultaneous runs of four chains for 10^7^ generations, sampling trees every 10^3^ generations. The ‘burn-in’ determined by stationarity of lnL assessed with Tracer v.1.5 (http://beast.bio.ed.ac.uk/Tracer) was set for the first 25% of the trees sampled, and a consensus topology and nodal support estimated as posterior probability values (Huelsenbeck *et al*., 2001) were calculated from the remaining trees. Phylogenetic trees were visualised and finalised in FigTree v. 1.4.4 (http://tree.bio.ed.ac.uk/software/figtree/.)).

## Results

### Prevalence of *Diplostomum phoxini* in the intermediate hosts

A total of 1599 *Ampullaceana balthica* representing 41 distinct individual samples (i.e. collected at a given place and date, as per the definition of Bush *et al*., 1997) were collected in the River Ruhr at Neheim from May to December during 2016, 2017 and 2019, and examined for prepatent and patent infections with *Diplostomum* spp. Infections with *D. phoxini* were detected from July to November (prevalence range: 3.3–13.6%, see Online Resource Table S2 for details) with the largest number of infected snails and greatest prevalence being recorded in September (five samples). Although large samples were examined in May (12.v.–22.v.; *n* = 255) and June (7.vi.–25.vi.; *n* = 250), no snails infected with *D. phoxini* were found (see Online Resource Table S2).

All dissected *P. phoxinus* (*n* = 15) were infected with large numbers of metacercariae of *D. phoxini* (*n* > 100) located in the optical lobes of the brain.

### Molecular identification of the snail host

Representative *cox*1 (591–610 nt; *n* = 2), 28S (1048 nt; *n* = 1) and ITS2 (402 nt; *n* = 1) sequences were generated for the snail host *A. balthica*. The new *cox*1 sequences differed at two nucleotide (nt) positions (0.03%). The new 28S rDNA sequence was identical with a sequence for *A*. *balthica* originally identified as *R*. *ovata* (EF417136; see Sonnenberg *et al*., 2007) and differed at a single-nucleotide position from an isolate originating from Russia (MH168039; Aksenova *et al*., 2018). ITS2 sequence comparisons revealed differences at 0–3 nt positions between the present isolate and the data for *A*. *balthica* available on GenBank; the new ITS2 sequence was identical with sequences for a total of 55 isolates of *A*. *balthica* originating from Belgium, Iceland, Norway and the UK.

Molecular identification of the snail host was further carried out on a *cox*1 alignment corresponding to the clade representing the subfamily Amphipepleinae Pini, 1877 in Aksenova *et al*. (2018) (36 species; 43 sequences; 636 nt) using *Galba truncatula* (O.F. Müller) (Lymnaeidae) as the outgroup. As shown in the NJ tree in Online Resource Fig. S1, the two newly generated *cox*1 sequences fell within the strongly supported clade of *Ampullaceana* spp. and clustered with five sequences for *A*. *balthica* originating from Europe and Asia with high support, thus confirming their identification based on morphology and the reclassification of *Radix balthica* to *Ampullaceana* (see Aksenova *et al*., 2018).

### Molecular characterization of *Diplostomum phoxini*

Partial *cox*1 sequences (349–407 nt) of *D*. *phoxini* were generated for a total of nine isolates (seven cercarial and two metacercarial), representing eight haplotypes (Table 1). Genetic divergence between seven cercarial and one metacercarial isolate and the two isolates of *D. phoxini* sequenced from Norway (see Soldánová *et al*., 2017) ranged between 0% and 1.2% (0–5 nt difference), whereas one newly sequenced metacercarial isolate (MZ615639) exhibited considerable divergence (2.2–3.1%, 9–12 nt difference) in the comparisons with the remaining isolates of *D*. *phoxini* from River Ruhr. A comparison with the recently published sequences for *D. phoxini* from *P. phoxinus* in Finland and Russia (Lebedeva *et al*., 2021) revealed an overall range for genetic divergence of 0–1.3%, excluding the most divergent haplotype sequenced from River Ruhr (MZ615639) and one most divergent haplotype sequenced from River Varzuga, Russia (MT982208: 3.5–4.8%; the upper limit represents the divergence between these two most divergent haplotypes). A total of 15 haplotypes were identified among the isolates sampled in Europe, including six novel haplotypes from the present material, six haplotypes from the material of Lebedeva *et al*. (2021) and one haplotype (KY513185) reported from Norway by Soldánová *et al*. (2017). One haplotype (MZ615634) was shared with an isolate ex *P. phoxinus* from Lake Ovre Heimdalsvatnet, Norway (KY513186; Soldánová *et al*., 2017) and an isolate ex *P. phoxinus* from River Uksa, Russia (MT982204; Lebedeva *et al*., 2021) and one haplotype was represented by two cercarial isolates from the River Ruhr (MZ615631 and MZ615632). Genetic divergence between *D. phoxini* and the species of the *D. baeri* complex ranged between 6.9% and 12.3% (Table 2).

**Table 1.** Summary data for isolates of *Diplostomum phoxini* and *Ampullaceana balthica* from the River Ruhr at Neheim (Germany) used for generation of the new *cox*1, ITS1-5.8S-ITS2 and 28S rDNA (domains D1–D3) sequences

Species	Site	Isolate	Life-cycle stage	GenBank ID (*cox*1/ITS1-5.8S-ITS2/28S)
*D. phoxini* ex *A. balthica*	B1	DphoAbal1	C	MZ615631
	B0	DphoAbal2	C	MZ615632
	B1	DphoAbal3	C	MZ615633
	B0	DphoAbal4	C	MZ615634/MZ616379^a^
	B0	DphoAbal5	C	MZ615635
	B2	DphoAbal6	C	MZ615636
	B2	DphoAbal7	C	MZ615637
*D. phoxini* ex *P. phoxinus*	B4	DphoPpho1	M	MZ615638/MZ616381/MZ616380
	B4	DphoPpho2	M	MZ615639/MZ616382^b^
*A. balthica*	B1	AbalRuhr1	A	MZ615629/MZ616378^c^/MZ616383
	B0	AbalRuhr1	A	MZ615630

*Abbreviations*: A, adult; C, cercaria; M, metacercaria.

a 28S.

b ITS1-5.8S-ITS2.

c ITS2 only.

**Table 2.** Percent interspecific genetic divergence (p-distance model) for *D. phoxini* compared with the species/lineages of the *D. baeri* species complex based on all *cox*1 sequences available on GenBank (retrieved on 29 June 2021)

Species/Lineage	*n*	Divergence (%)
*D. adamsi* (syn. *D. baeri sensu* Galazzo *et al*., 2002)	979	8.7–11.6
*Diplostomum* sp. 5 of Locke *et al*. (2010*a*)	11	8.8–9.5
*Diplostomum* sp. 6 of Locke *et al*. (2010*a*)	44	8.3–10.0
*Diplostomum* sp. 7 of Locke *et al*. (2010*a*)	176	7.5–9.6
*Diplostomum* sp. Lineage 3 of Blasco-Costa *et al*. (2014)	451	9.1–12.3
*Diplostomum* sp. Lineage 4 of Blasco-Costa *et al*. (2014)	473	8.7–11.3
*Diplostomum* sp. Lineage 5 of Blasco-Costa *et al*. (2014)	286	6.9–9.2
*Diplostomum* sp. of Lebedeva *et al.* (2021)	144	9.1–11.1

*Abbreviation: n*, number of pairwise comparisons

Additionally, two partial 28S (1214–1223 nt) and two complete ITS1-5.8S-ITS2 (1046–1049 nt) rDNA sequences were generated for representative cercarial and metacercarial isolates of *D*. *phoxini* (Table 1). The newly generated 28S sequences were identical and differed at six positions from the published sequence for *D*. *phoxini* by Olson *et al*. (2003).

### Morphological characterisation of *Diplostomum phoxini*

#### Description of the cercaria

**Fig. 1.**
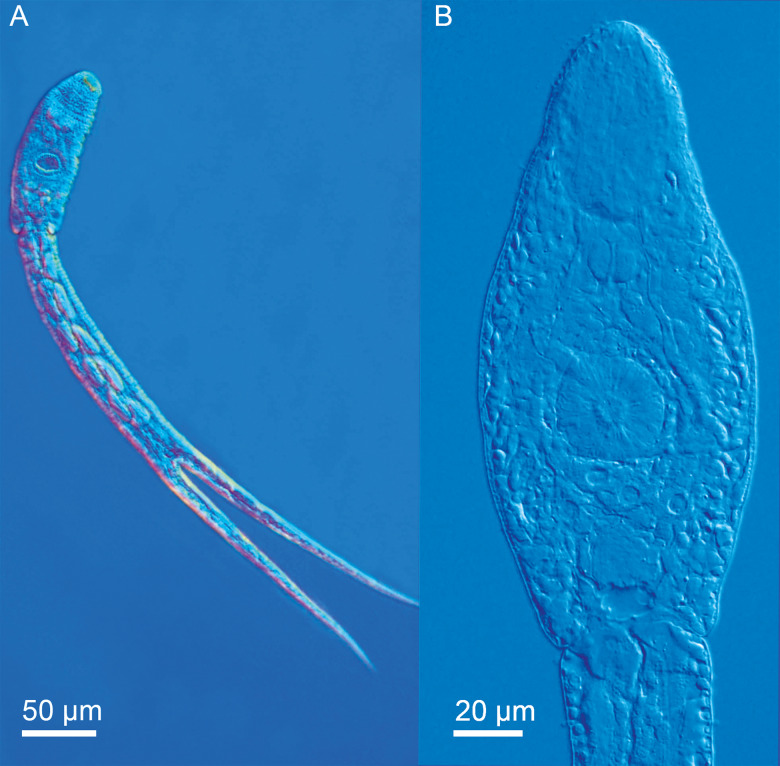
Cercaria of *Diplostomum phoxini* ex *Ampullaceana balthica* (light microscopy). A, Resting position; B, Cercarial body.

**Fig. 2.**
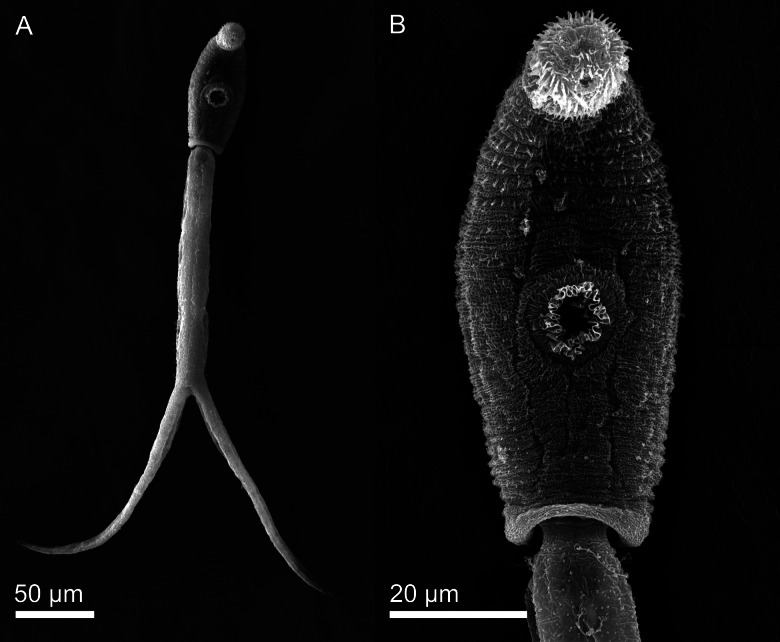
Cercaria of *Diplostomum phoxini* ex *Ampullaceana balthica* (scanning electron microscopy), ventral view. A, Entire cercaria; B, Cercarial body.

**Fig. 3.**
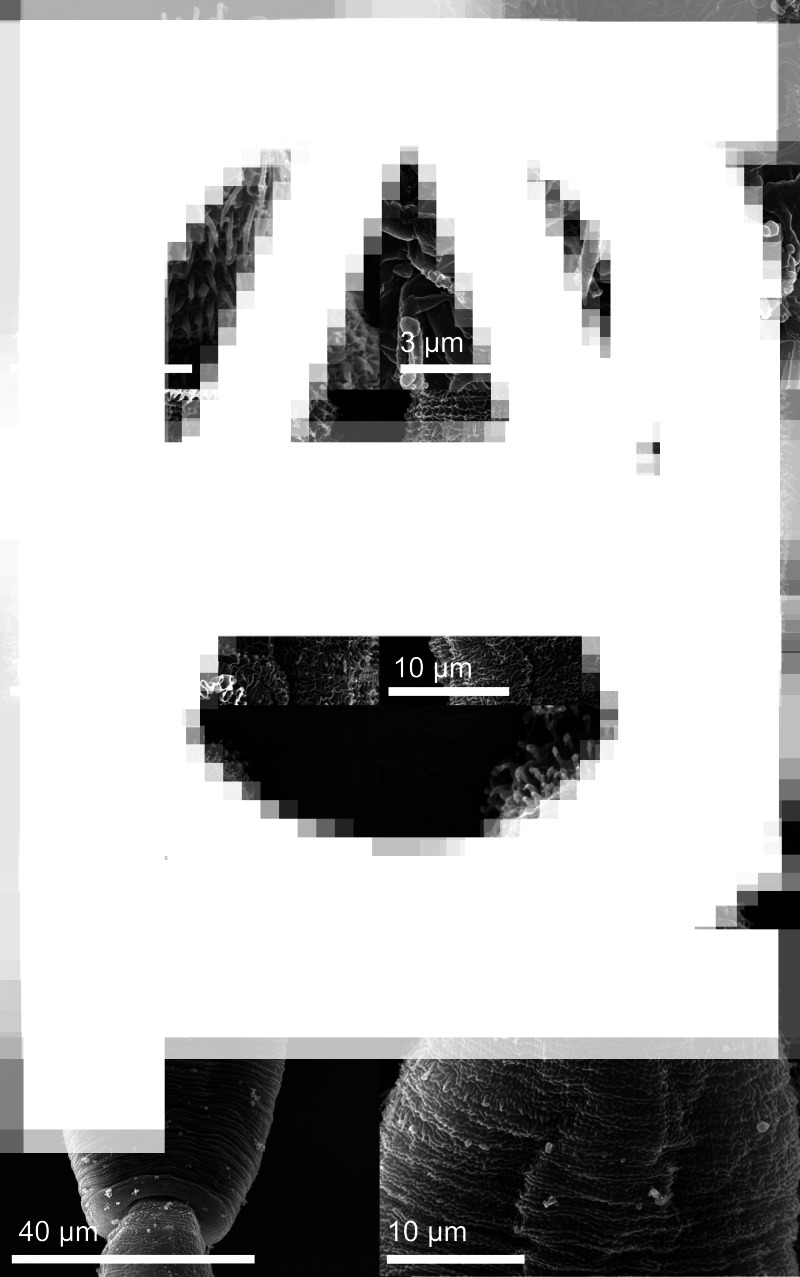
Cercaria of *Diplostomum phoxini* ex *Ampullaceana balthica* (scanning electron microscopy), ventral view. A, Anterior organ, ventral view; B, Pre-oral spines, apical view; C, Transverse rows of tegumental spines on the body, ventral view; D, Ventral sucker with a well-developed undulating membrane, ventral view; E, Cercarial body, dorsal view; F, Anterior part of cercarial body, dorsal view at a higher magnification.

[Based on 40 live specimens; (Figs 1–3; Table 3 and Online Resource Table S3)] Body elongate-oval, 138–154 × 37–50, shorter than tail stem (TSL/BL = 1.34–1.62). Anterior organ elongate-oval, 52–59 × 27–31. Ventral sucker subspherical, with small undulating membrane (Figs 2 and 3), just post-equatorial, 30–34 × 30–33; width exceeding width of anterior organ (AOW/VSW = 0.84–0.94). Mouth opening ventro-subterminal; prepharynx long, narrower in anterior organ; pharynx round to elongate-oval, muscular, 11–14 × 12–16, followed by short oesophagus bifurcating anterior to ventral sucker; intestinal caeca well developed, terminating almost at posterior extremity of body. Penetration gland-cells two pairs with fine granular content, similar in size, posterior to ventral sucker, overlap caeca, posterior pair not reaching extremities of caeca; ducts open antero-laterally to mouth, two on either side. Anlagen of reproductive organs a compact mass of small cells just anterior to excretory vesicle. Tail stem 212–226 long, 29–37 wide at base, nearly as long as furcae, 212–239 long, 13–24 wide at base (TSL/FL = 0.95–1.06), with six pairs of caudal bodies with slightly irregular margins along excretory duct. Furcae 212–239 long, 13–24 wide at base, without fin-folds. Excretory vesicle small, V-shaped, with round stem; caudal excretory duct passes through tail stem; excretory pores at mid-length of furcae.

**Table 3.** Comparative data for the cercariae of *Diplostomum phoxini* and species of the *D. baeri* species complex

Species/Feature	*D. phoxini*				*D. baeri* Dubois, 1937	*Diplostomum* sp. Lineage 4 of Blasco-Costa *et al.* (2014)
Source	Present study	Arvy and Buttner (1954)	Rees (1957)	Dönges (1969*a*)	Niewiadomska and Kiselienė (1994)	Faltýnková *et al.* (2014)
Relation BL-TSL-FL	BL < TSL = FL	BL < TSL > FL	BL < TSL > FL	BL < TSL > FL	BL < TSL < FL	BL < TSL = FL
Relation VSW/AOW	VSW > AOW	VSW > AOW	VSW > AOW	VSW > AOW	VSW = AOW	VSW < AOW
No. of pre-oral spines in the median group	10	–	5	–	7–11	8
No. of pre-oral spines in each lateral group	Absent	–	Absent	–	Absent	Absent
No. of post-oral rows of spines	7	–	7	–	7–9	5–6
No. of transverse rows of spines on body	8 (rows 1–5 complete ventrally, rows 6–8 incomplete ventrally; only row 1 complete dorsally)	–	8 (rows 1–5 complete ventrally, rows 6–8 incomplete ventrally; only rows 1–2 complete dorsally)	8	10	9
No. of spine rows on ventral sucker	2	–	2	2	3	3
No. of spines on ventral sucker	40 per row (*c*.80 in total)	28	36 per row (*c*.72 in total)	48 per row (*c*.96 in total)	90–130	38 per row (*c*.120 in total)
Penetration gland-cells	Large, do not cover ends of caeca	Small, do not cover ends of caeca	Large, do not cover ends of caeca	–	Large, cover ends of caeca	Large, cover ends of caeca
Spines on tail stem	Present	–	–	–	Absent	Present
Spines on furcae	Present	–	–	–	Absent	Present
Resting position	Tail stem straight	–	Tail stem straight	Tail stem straight	Tail stem straight	Tail stem straight

*Body armature*: Pre-oral spines arranged in a single median group of 10 spines in two rows, anterior row comprised of four spines; two central larger than the lateral; lateral groups of pre-oral spines lacking. Post-oral spines more robust than spines on body, in seven alternate rows encircling body to about mid-level of anterior organ; spines in first two rows much larger than remaining spines, all of similar size. Wide zone of smaller, less dense, irregularly dispersed spines present posterior to post-oral spines. Transverse rows of spines 8, extending to about mid-level of ventral sucker ventrally. Rows 1–5 complete ventrally; rows 6–8 incomplete ventrally; only row 1 complete dorsally. Two ventro-lateral non-confluent fields of smaller spines present in posterior body third. Ventral sucker armed with two rows of spines (*c*. 40 spines per row). Tail stem and furcae armed with minute spines; spines along tail stem in two ventral and two dorsal bands with two medio-lateral bands consisting of small, irregularly dispersed scale-like spines. Spines on furcae in one medial band laterally, consisting of 1–3 scale-like spines, size and density of spines decreasing distally.

*Resting position*: Tail stem straight, body slightly bent ventrally.

#### Description of the metacercaria

**Fig. 4.**
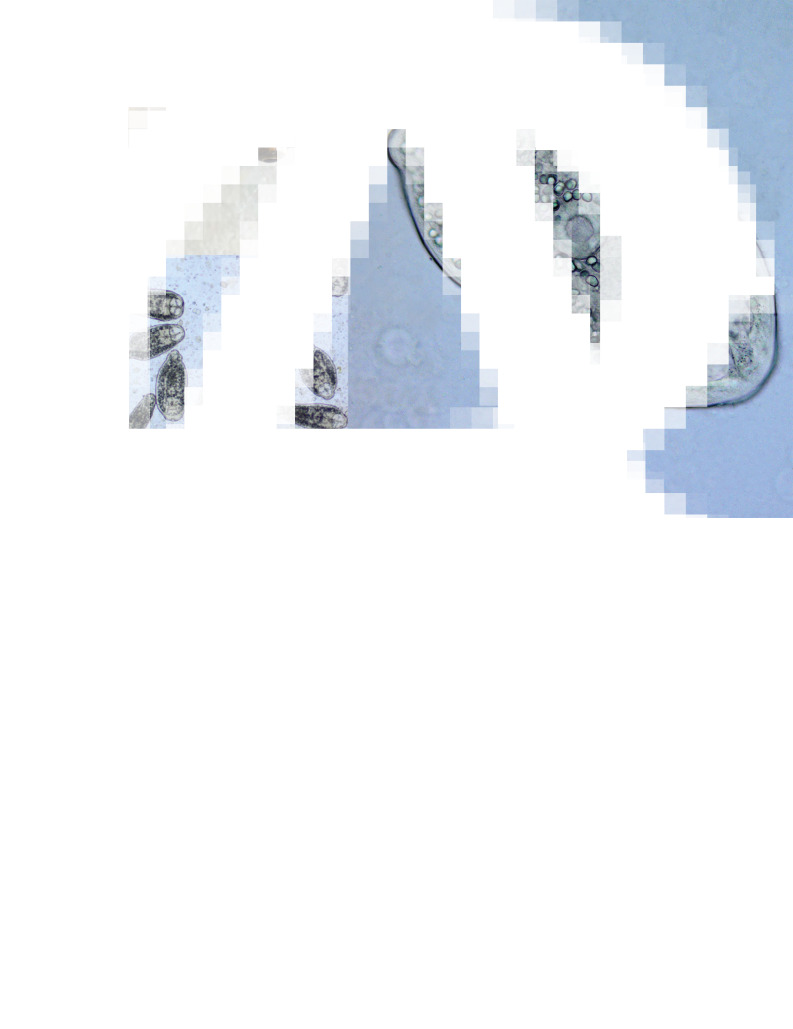
*Phoxinus phoxinus* (A) and live metacercariae of *Diplostomum phoxini* (B, C).

[Based on 20 live specimens from the optical lobes of the brain of *Phoxinus phoxinus*; Fig. 4; Online Resource Table S4] Body elongate-oval, 326–411 (358), with maximum width just anterior to ventral sucker, 145–227 (186). Oral sucker subterminal, subspherical, 38–56 × 36–50 (49 × 44). Pseudosuckers two, contractile, small-sized, 28–42 × 20–40 (34 × 30). Prepharynx indistinct; pharynx muscular, elongate-oval, 18–39 × 12–22 (31 × 18); oesophagus very short, bifurcates close posterior to pharynx; intestinal caeca narrow, encroach holdfast organ and terminate blindly at mid-level of excretory vesicle. Ventral sucker subspherical, 34–48 × 43–54 (43 × 47), similar in size to oral sucker or slightly larger [VSW/OSW = 1.0–1.1 (1.1)], at mid-body length or slightly posterior. Holdfast organ massive, 47–95 × 78–102 (71 × 91), bi-partite with median slit, transversely oval, contiguous with ventral sucker and excretory vesicle. Excretory vesicle large, conspicuous, V-shaped; reserve excretory system of diplostomid type; excretory concretions predominantly large, 345–579 (454) in number, grouped into two lateral and one median fields. Hindbody short, 24–53 (42).

#### Remarks

The present detailed descriptions expand the known range of variation of the metrical features and provide morphological detail that will facilitate the morphological identification of the larval stages of *D. phoxini*. The cercaria of *D*. *phoxini* resembles the known cercariae of the *D. baeri* species complex in the lack of the lateral group of pre-oral spines and the resting position with a straight tail stem but differs in the following species-specific features: (i) BL < TSL ⩾ FL; (ii) VSW > AOW; (iii) eight transverse rows of spines on the body; (iv) two rows of spines on the ventral sucker; (v) penetration gland-cells not covering ends of caeca (Table 3). Although the metrical data exhibit overlapping ranges for some features, the cercaria of *D. phoxini* can be distinguished from the cercaria of both *D. baeri sensu* Niewiadomska and Kiselienė (1994) and *Diplostomum* sp. Lineage 4 of Blasco-Costa *et al*. (2014) in having on average smaller body, shorter tail and furcae, a narrower apical organ; the tail in *D. phoxini* is also much longer than the body (Faltýnková *et al*., 2014; see Online Resource Table S3 for details).

The metacercaria of *D. phoxini* exhibits overlapping ranges for the metrical data with *Diplostomum* sp. Lineages 3–5 of Blasco-Costa *et al*. (2014) but the means for the latter species-level lineages are greater (Online Resource Table S4; see also Faltýnková *et al*., 2014). Comparisons with the metacercariae measured by Lebedeva *et al*. (2021) revealed an overall agreement for the metrical data except for the somewhat smaller body dimensions and the greater number of excretory concretions (Online Resource Table S4). Both differences are due to the fact that Lebedeva *et al*. (2021) examined fixed material; this may have led to misinterpretations of excretory concretions.

Parasitism in a specific second intermediate host (*P. phoxinus*) can be also used to distinguish *D. phoxini* from *Diplostomum* sp. Lineage 4 of Blasco-Costa *et al*. (2014), the only species of the *D. baeri* complex with a European distribution which was also recorded in the brain of *Gasterosteus aculeatus*.

### Phylogenetic analyses

The newly generated *cox*1 sequences were analysed together with all published sequences for *Diplostomum* spp. (1203 sequences; 407 nt). The neighbour-joining analysis depicted 44 species/species-level lineages of *Diplostomum* with typically maximum or very high support (see Table 4 and Online Resource Fig. S2); these included seven taxa represented by singletons: *Diplostomum* sp. 5 and *Diplostomum* sp. 8 of Locke *et al*. (2010*a*); *Diplostomum* sp. 11 of Locke *et al*. (2015); *Diplostomum* sp. A of Gordy and Hanington (2019), *Diplostomum* spp. A, B and C of Kudlai *et al*. (2017).

**Table 4.** Species and species-level lineages of *Diplostomum* with a re-identification of some isolates (GenBank data as of 29 June 2021; see the neighbour-joining tree based on all available sequences in Online Resource Fig. S2 and Online Resource Table S5 for details)

Species/Lineage	Reference	Distribution	Re-identified isolates [Reference]	Isolates with updated nomenclature [Reference]
*D. ardeae* Dubois, 1969	Locke *et al*. (2015, 2020)	Canada, Puerto Rico		
*D. adamsi* (syn. *D. baeri sensu* Galazzo *et al*., 2002)	Moszczynska *et al*. (2009); Locke *et al*. (2010a, 2010*b*, 2015); Ubels *et al*. (2018)	Canada, USA	*Diplostomum baeri* complex sp. LIN2 (isolates MGC1740, MGC1824, MGC1861, MGC2242) ex *Ladislavella elodes* (Canada) (MH368850-MH368853) [Gordy and Hanington, 2019]	
			*Diplostomum* aff. *baeri* LIN2 (isolate MGC190) ex *Ladislavella elodes* (Canada) (KT831353) [Gordy and Hanington, 2019]	
*D. huronense* (La Rue, 1927)	Moszczynska *et al*. (2009); Locke *et al*. (2010*a*, 2010*b*, 2015)	Canada		
*D. indistinctum* (Guberlet, 1922)	Moszczynska *et al*. (2009); Locke *et al.* (2010*a*, 2010*b*, 2015; Gordy *et al*. (2016)	Canada		
*D. lunaschiae* Locke, Drago, Núñez, Rangel e Souza & Takemoto, 2020	Locke *et al*. (2020)	Argentina, Brazil		
*D. mergi* Lineage 2 of Georgieva *et al*. (2013)	Georgieva *et al*. (2013); Selbach *et al*. (2015); Kudlai *et al*. (2017); Locke *et al.* (2015)	China, Germany, Slovakia, Hungary		*D. mergi* ex *Radix auricularia* (Germany) (JX986874-JX986876) [Georgieva *et al*., 2013]^a^
				*D. mergi* ex *Radix auricularia* (Germany) (KR149513-KR149523) [Selbach *et al*., 2015]^b^
*D. mergi* Lineage 3 of Georgieva *et al*. (2013)	Georgieva *et al*. (2013); Selbach *et al*. (2015)	Germany		*D. mergi* ex *Salmo trutta* and *Gobio gobio* (Germany) (JX986877-JX986885) [Georgieva *et al*., 2013]^c^
				*D. mergi* (isolates RaHe17-RaHe19) ex *Radix auricularia* (Germany) (KR149524-KR149527) [Selbach *et al*., 2015]^d^
*D. mergi* Lineage 4 of Selbach *et al*. (2015)	Selbach *et al*. (2015)	China, Germany	*D. mergi* (China) (KY271543) [Dang (unpublished)]	*D. mergi* (isolate RaHe20) ex *Radix auricularia* (Germany) (KR149528) [Selbach *et al*., 2015]^e^
*D. parviventosum* Dubois, 1932^f^	Selbach *et al*. (2015)	Germany		*D. mergi* (isolate RAH1) ex *Radix auricularia* (Germany) (JX986873) [Georgieva *et al*., 2013]^g^
*D. phoxini* (Faust, 1919)	Soldánová *et al*. (2017); Lebedeva *et al*. (2021); present study	Finland, Germany, Norway, Russia		
*D. pseudospathaceum* Niewiadomska, 1984	Georgieva *et al*. (2013); Behrmann-Godel (2013); Pérez-del-Olmo *et al*. (2014); Locke *et al*. (2015); Kudlai *et al*. (2017); Enabulele *et al*. (2018)	Czech Republic, Germany, Hungary, Poland, Romania, Slovakia, Spain, UK		
*D. spathaceum* (Rudolphi, 1819)	Georgieva *et al*. (2013); Blasco-Costa *et al*. (2014); Pérez-del-Olmo *et al*. (2014); Locke *et al*. (2015); Kudlai *et al*. (2017); Dang *et al*. (unpublished)	China, Croatia, Czech Republic, Germany, Hungary Iceland, Iraq, Italy, Poland, Romania, Slovakia, Spain	*D. paracaudum* (isolate RA155) ex *Radix auricularia* (Germany) (JQ639176) [Behrmann-Godel, 2013]	
*Diplostomum* sp. 1 of Moszczynska *et al*. (2009)	Moszczynska *et al*. (2009); Locke *et al*. (2010*a*, 2010*b*, 2015); Rudko *et al*. (2018); Gordy and Hanington (2019); Ubels *et al*. (2018)	Canada, USA		
*Diplostomum* sp. 2 of Moszczynska *et al*. (2009)	Moszczynska *et al*. (2009); Locke *et al*. 2010*a*, 2010*b*)	Canada, USA		
*Diplostomum* sp. 3 of Moszczynska *et al*. (2009)	Moszczynska *et al*. (2009); Locke *et al*. (2010*a*, 2010*b*, 2015); Gordy *et al*. (2016); Gordy and Hanington (2019); Ubels *et al*. (2018)	Canada, USA	*D. baeri* (isolate 49D-R2) ex *Perca flavescens* on GenBank but the host is given as *Luxilus cornutus* in Fig. S1 (USA) (MF142178) [Ubels *et al*., 2018]	
*Diplostomum* sp. 4 of Moszczynska *et al*. (2009)	Moszczynska *et al*. (2009); Locke *et al*. (2010*a*, 2010*b*), Gordy and Hanington (2019); Rudko *et al*. (2018); Ubels *et al*. (2018)	Canada, USA	*D. baeri* (isolate 55D-T1) ex *Luxilus cornutus* on GenBank but the host is given as *Perca flavescens* in Fig. S1 (USA) (MF142161) [Ubels *et al*., 2018]	
*Diplostomum* sp. 5 of Locke *et al*. (2010a)	Locke *et al*. (2010*a*)	Canada		
*Diplostomum* sp. 6 of Locke *et al*. (2010a)	Locke *et al*. (2010*a*, 2015)	Canada		
*Diplostomum* sp. 7 of Locke *et al*. (2010a)	Locke *et al*. (2010*a*, 2015)	Canada		
*Diplostomum* sp. 8 of Locke *et al*. (2010a)	Locke *et al*. (2010*a*)	Canada		
*Diplostomum* sp. 9 of Locke *et al*. (2010a)	Locke *et al*. (2010*a*, 2015)	Canada		
*Diplostomum* sp. 10 of Locke *et al*. (2015)	Locke *et al*. (2015)	Canada		
*Diplostomum* sp. 11 of Locke *et al*. (2015)	Locke *et al*. (2015)	Canada		Diplostomidae gen. SL sp. 1 SAL-2010 (isolate Di.BR.Bo.Rp.2.1) ex *Rana pipiens* (Canada) (HM064650) [Locke *et al*., 2010*b*]
*Diplostomum* sp. 12 of Locke *et al*. (2015)	Locke *et al*. (2015)	Canada, USA		
*Diplostomum* sp. 13 of Locke *et al*. (2015)	Locke *et al*. (2015)	Canada, USA	*Diplostomum* sp. C MAG-2016 ex *Ladislavella elodes* (Canada) (KT831360, KT831378, KT831382) [Gordy and Hanington, 2019]	
			*Diplostomum* sp. C MAG-2019 ex *Ladislavella elodes* and *Planorbella trivolvis* (Canada) (MH368933-MH368941) [Gordy and Hanington, 2019]	
*Diplostomum* sp. 14 of Locke *et al*. (2015)	Locke *et al*. (2015); Hoogendoorn *et al*. (2020)	China, Iraq, South Africa		
*Diplostomum* sp. 15 of Locke *et al*. (2015)	Locke *et al*. (2015)	China		
*Diplostomum* sp. 16 of Locke *et al*. (2015)	Locke *et al*. (2015); Hoogendoorn *et al*. (2020)	Iraq, South Africa		
*Diplostomum* sp. 17 of Locke *et al*. (2015)	Locke *et al*. (2015)	Canada		
*Diplostomum* sp. 18 of Locke *et al*. (2015)	Locke *et al*. (2015)	Canada	*Diplostomum* sp. B MAG-2019 (isolate MGC2308) ex *Ladislavella elodes* (Canada) (MH368932) [Gordy and Hanington, 2019]	
*Diplostomum* sp. 19 of Locke *et al*. (2015)	Locke *et al*. (2015)	Canada, USA		*Diplostomum* sp. BOLD ACK9826 ex *Osmerus mordax* (Canada) (KM538089) [Van Steenkiste *et al*. (2015)]
*Diplostomum* sp. Lineage 2 of Blasco-Costa *et al*. (2014)^h^	Blasco-Costa *et al*. (2014); Faltýnková *et al*. (2014)	Iceland		
*Diplostomum* sp. Lineage 3 of Blasco-Costa *et al*. (2014)^h^	Blasco-Costa *et al*. (2014); Faltýnková *et al*. (2014); Soldánová *et al*. (2017)	Germany, Iceland, Norway, UK	*D. baeri* (isolates LF1-LF10) ex *Salmo trutta* (UK) (MT311204-MT311213) [Landeryou *et al*., 2020]	*D. baeri* ex *Salmo trutta* and *Gobio gobio* (Germany) (JX986859-JX986872) [Georgieva *et al*., 2013]^i^
*Diplostomum* sp. Lineage 4 of Blasco-Costa *et al*. (2014)^h^	Blasco-Costa *et al*. (2014); Faltýnková *et al*. (2014); Locke *et al.* (2015); Kuhn *et al.* (2015); Soldánová *et al*. (2017)	Germany, Iceland, Italy, Norway, Romania, UK	*D. baeri* ex *Perca fluviatilis* (Germany) (JQ639180-JQ639195) [Behrmann-Godel, 2013]^j^	
			*D baeri* (isolates Diplob2UistGa01-Diplob2UistGa04) ex *Gasterosteus aculeatus* (UK) (KX037874-KX037877) [Rahn *et al*., 2016]	
*Diplostomum* sp. Lineage 5 of Blasco-Costa *et al*. (2014)^h^	Blasco-Costa *et al*. (2014); Faltýnková *et al*. (2014); Locke *et al*. (2015); Soldánová *et al*. (2017)	Iceland, Norway		
*Diplostomum* sp. Lineage 6 of Blasco-Costa *et al*. (2014)^h^	Blasco-Costa *et al*. (2014); Faltýnková *et al*. (2014); Kuhn *et al*. (2015); Soldánová *et al*. (2017)	Iceland, Norway, UK	*Diplostomum* sp. 6 AKR-2016 ex *G. aculeatus* ((isolates Diplolin6ICEGa02-Diplolin6ICEGa05; Diplolin6UistGa01-Diplolin6UistGa35) (Iceland, UK) (KX037902-KX037915; KX140051-KX140055) and *P. pungitius* (isolates Diplolin6UistPp01-Diplolin6UistPp03) (UK) KX037878-KX037880) [Rahn *et al*., 2016]	
*Diplostomum* sp. A of Gordy and Hanington (2019)	Gordy and Hanington (2019)	Canada		
*Diplostomum* sp. A of Kudlai *et al*. (2017)	Kudlai *et al*. (2017)	Slovakia		
*Diplostomum* sp. B of Kudlai *et al*. (2017)	Kudlai *et al.* (2017)	Slovakia		
*Diplostomum* sp. C of Kudlai *et al*. (2017)	Kudlai *et al*. (2017)	Slovakia		
*Diplostomum* sp. of Chibwana *et al*. (2013)	Chibwana *et al*. (2013); Hoogendoorn *et al*. (2020)	Nigeria, South Africa		
*Diplostomum* sp. (Japan) of Komatsu *et al*. (2019)	Komatsu *et al*. (2019)	Japan		
*Diplostomum* sp. Clade Q	Georgieva *et al*. (2013); Pérez-del-Olmo *et al*. (2014); Selbach *et al*. (2015); Locke *et al*. (2015)	Germany, Spain	*Diplostomum mergi* (RR43, RR45, RA97) ex *Rutilus rutilus* and *Radix auricularia* (Germany) (JQ639177-JQ639179) [Behrmann-Godel, 2013]	
*Diplostomum* sp. of Lebedeva et al. (2021)	Lebedeva et al. (2021)	Mongolia		

a *D. mergi* Lineage 2 (“*D. mergi* 2” and “Clade 3-2”) in Georgieva *et al*. (2013)

b *D. mergi* Lineage 2 in Selbach *et al*. (2015) and Kudlai *et al*. (2017)

c *D. mergi* Lineage 3 (“*D. mergi* 3” and “Clade 3-3”) in Georgieva *et al*. (2013)

d *D. mergi* Lineage 3 in Selbach *et al*. (2015)

e *D. mergi* Lineage 4 in Selbach *et al*. (2015)

f Member of the *D. mergi* species complex, indicated as *D. mergi* Lineage 1 in Georgieva *et al*. (2013)

g *D. mergi* Lineage 1 (“*D. mergi* 1” and “Clade 3-1”) in Georgieva *et al*. (2013)

h Lineages discovered in Iceland and characterised molecularly and morphologically by Blasco-Costa *et al*. (2014) and Faltýnková *et al*. (2014), respectively

i *D. baeri* Lineage 1 (“*D. baeri* 1”, “Clade 4-1” or “trout lineage”) in Georgieva *et al*. (2013)

j *D. baeri* Lineage 2 (“*D. baeri* 2”, “Clade 4-2” or “perch lineage”) in Georgieva *et al*. (2013)

The sequences for *D*. *phoxini* clustered in a strongly supported reciprocally monophyletic lineage associated with three lineages of the *D. baeri* species complex *sensu* Blasco-Costa *et al*. (2014): *Diplostomum* spp. 5 and 6 of Locke *et al*. (2010*a*), and *Diplostomum* sp. Lineage 5 of Blasco-Costa *et al*. (2014). The sequences for the second lineage of *Diplostomum* sp. from the brain of *P. phoxinus* discovered in Mongolia by Lebedeva *et al*. (2021) also formed a strongly supported monophyletic clade (Online Resource Fig. S2).

**Fig. 5.**
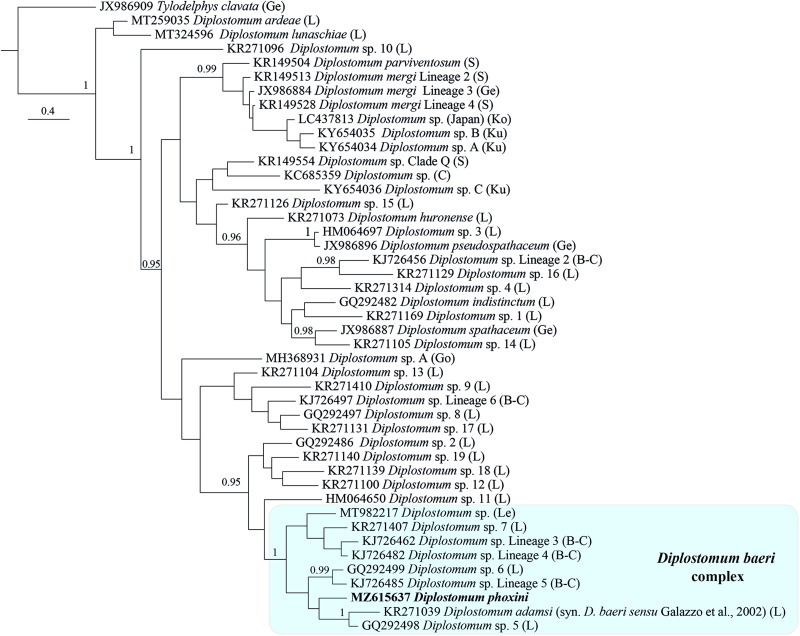
Phylogram from Bayesian inference (BI) analysis of the *cox*1 sequence alignment (407 nt) for 44 species/species-level lineages of *Diplostomum*. Outgroup: *Tylodelphys clavata*. Nodal support is given as posterior probabilities; only values ⩾ 0.95 are shown. The scale-bar indicates the expected number of substitutions per site. The shaded rectangle indicates the content of the *Diplostomum baeri* species complex inferred from the present study. *Abbreviations*: B-C, Blasco-Costa *et al*. (2014); C, Chibwana *et al*. (2013); Ge, Georgieva *et al*. (2013); Go, Gordy and Hanington (2019); L, Locke *et al*. (2010*a*, 2010*b*, 2015, 2020); Le, Lebedeva *et al*. (2021); Ko, Komatsu *et al*. (2019); Ku, Kudlai *et al*. (2017); S, Selbach *et al*. (2015).

Bayesian inference phylogenetic reconstruction for representatives of the genus *Diplostomum* (currently comprising 44 species/species-level lineages, see Table 4) depicted a composition of the *D. baeri* species complex similar to that in Blasco-Costa *et al*. (2014) (Fig. 5). The only differences are the addition of *D. phoxini* and *Diplostomum* sp. of Lebedeva *et al*. (2021) and the exclusion of *Diplostomum* sp. 2 of Moszczynska *et al*. (2009). There was support for a close association with the *D. baeri* complex for 5 lineages: *Diplostomum* spp. 2, 12, 18 and 19 of Locke *et al*. (2015) forming a cluster, albeit with poor support, plus the singleton ex *Rana pipiens* (*Diplostomum* sp. 11). Within the *D. baeri* species complex, there was a strongly supported sister-group relationship between: (i) *Diplostomum* sp. Lineage 5 of Blasco-Costa *et al*. (2014) and *Diplostomum* sp. 6 of Locke *et al*. (2010*a*); and (ii) *D. baeri sensu* Galazzo *et al*. (2002) and *Diplostomum* sp. 5 of Locke *et al*. (2010*a*).

The present phylogenetic hypothesis for *Diplostomum* spp. depicted two additional well supported clusters: a group of 10 lens-infecting species/lineages containing the type-species of the genus, *D. spathaceum*; and the species/lineages of the *D. mergi* species complex *sensu* Selbach *et al*. (2015) which also included one additional lineage from Japan (see Komatsu *et al*., 2019) and two singletons from River Danube in Slovakia (see Kudlai *et al*., 2017) (Fig. 5). The relationships of the remaining species/lineages remained unresolved.

**Fig. 6:**
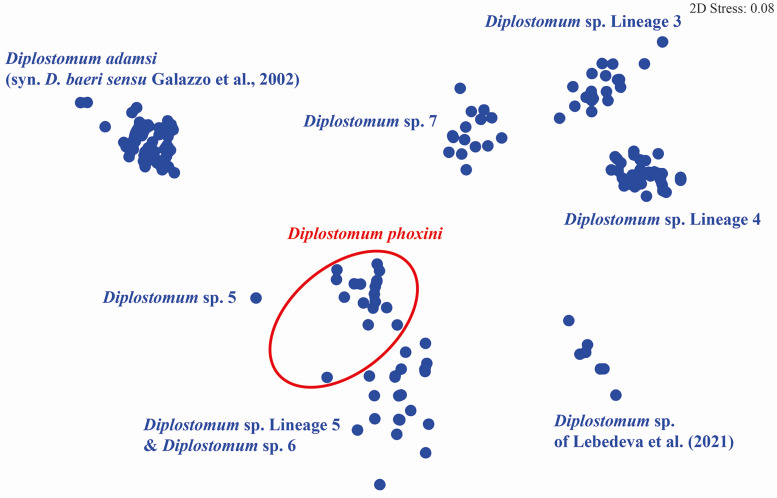
Non-metric multidimensional scaling ordination plot derived from the raw pairwise distances (p-distance) calculated for the species/lineages of the *D. baeri* complex based on the *cox*1 dataset.

By means of raw pairwise interspecific divergence, the species/lineages of the *D. baeri* species complex appear well differentiated except for the two loose groups indicated by ellipses in the NMDS plot (Fig. 6) comprising *D. phoxini* and *Diplostomum* sp. Lineage 5 of Blasco-Costa *et al*. (2014) + *Diplostomum* sp. 6 of Locke *et al*. (2010*a*) (range for the latter two lineages: 3.5–5.5%) (see ranges for *D. phoxini* in Table 2). Importantly, comparisons of the isolates of *D. baeri sensu* Galazzo *et al*. (2002) sampled in North America with the species/lineages of the *D. baeri* species complex revealed levels of genetic divergence (7.7–15.3%, see Table 5 for details) within the range reported for distinct species/lineages of *Diplostomum* (4.2–16.4%, see Georgieva *et al*., 2013; 4.3–14.7%, see Selbach *et al*., 2015). The intraspecific divergence for these isolates was low (0–1.3%; based on 3872 pairwise comparisons).

**Table 5.** Percent interspecific genetic divergence (p-distance model) for *D. adamsi* (syn. *D. baeri sensu* Galazzo *et al*., 2002) sampled in North America compared with the species/lineages of the *D. baeri* species complex based on all *cox*1 sequences available on GenBank (retrieved on 29 June 2021)

Species/Lineage	*n*	Divergence (%)
*D. phoxini*	1602	8.7–12.1
*Diplostomum* sp. 5 of Locke *et al*. (2010*a*)	89	7.7–9.2
*Diplostomum* sp. 6 of Locke *et al*. (2010*a*)	356	9.8–13.5
*Diplostomum* sp. 7 of Locke *et al*. (2010*a*)	1424	9.0–12.0
*Diplostomum* sp. Lineage 3 of Blasco-Costa *et al*. (2014)	3649	10.3–14.5
*Diplostomum* sp. Lineage 4 of Blasco-Costa *et al*. (2014)	3827	11.2–15.3
*Diplostomum* sp. Lineage 5 of Blasco-Costa *et al*. (2014)	1780	9.3–13.1
*Diplostomum* sp. of Lebedeva *et al.* (2021)	712	10.2–12.9

*Abbreviation*: *n*, number of pairwise comparisons

### Re-classification and an updated nomenclature for *Diplostomum* spp.

The present phylogenetic analyses helped update the nomenclature of a total of 144 sequenced isolates of *Diplostomum* spp. (see Table 4 for a summary and Online Resource Table S5 for a detailed list of all isolates; these are also indicated in the tree in Online Resource Fig. S2). Of these, we updated the species/lineage definitions with links to the GenBank accession numbers for 46 isolates (indicated in blue in Online Resource Fig. S2 and Table S5) and re-classified 98 isolates (indicated in red in Online Resource Fig. S2 and Table S5) assigned to 11 lineages as follows:

- *D. adamsi* (syn. *D. baeri sensu* Galazzo *et al*., 2002): reported as “*Diplostomum baeri* LIN2” by Gordy and Hanington (2019) and annotated on GenBank as “*Diplostomum baeri* complex sp. LIN2”and “*Diplostomum* aff. *baeri* LIN2”;
- *D. mergi* Lineage 4 of Selbach *et al*. (2015): annotated as *D. mergi* on GenBank by Dang (unpublished sequence); this lineage is also not correctly annotated by Selbach *et al*. (2015);
- *D. spathaceum*: reported and annotated on GenBank as *D. paracaudum* by Behrmann-Godel (2013); this isolate has been re-classified by Georgieva *et al*. (2013);
- *Diplostomum* sp. 3 of Moszczynska *et al*. (2009): reported and annotated on GenBank as *D. baeri* by Ubels *et al*. (2018);
- *Diplostomum* sp. 4 of Moszczynska *et al*. (2009): reported and annotated on GenBank as *D. baeri* by Ubels *et al*. (2018);
- *Diplostomum* sp. 13 of Locke *et al*. (2015): reported and annotated on GenBank as “*Diplostomum* sp. C” by Gordy and Hanington (2019);
- *Diplostomum* sp. 18 of Locke *et al*. (2015): reported and annotated on GenBank as “*Diplostomum* sp. B” by Gordy and Hanington (2019);
- *Diplostomum* sp. Lineage 3 of Blasco-Costa *et al*. (2014): reported as *D. baeri* by Landeryou *et al*. (2020);
- *Diplostomum* sp. Lineage 4 of Blasco-Costa *et al*. (2014): reported and annotated on GenBank as *D. baeri* by Behrmann-Godel (2013) (assigned to the “perch” lineage or “*D. baeri* ” by Georgieva *et al*., 2013); reported as “*D. baeri* 2” and annotated on GenBank as *D. baeri* by Rahn *et al*. (2016);
- *Diplostomum* sp. Lineage 6 of Blasco-Costa *et al*. (2014): reported as “*D*. Lineage 6” but annotated on GenBank as *Diplostomum* sp. 6 by Rahn *et al*. (2016); and
- *Diplostomum* sp. Clade Q: reported and annotated on GenBank as *D. mergi* by Behrmann-Godel (2013); these isolates have been re-classified by Georgieva *et al*. (2013).

## Discussion

*Diplostomum phoxini* is a well-delimited species which differs from all described species of *Diplostomum* in the morphology of the adult stage, the strict host specificity to the second intermediate hosts (*P. phoxinus*) and the specific location in the fish brain. Nevertheless, in light of the expansive development of molecular studies on *Diplostomum* spp. and the current uncertainty in linking sequence data from larval stages to named species of this genus, it is desirable to describe the sequenced forms and thus anchor the molecular data to morphological reference (see e.g. Blasco-Costa *et al*., 2014; Faltýnková *et al*., 2014; Pérez-del-Olmo *et al*., 2014; Selbach *et al*., 2015). Our study thus anchors the molecular data to detailed descriptions of the larval stages and provides molecular evidence for the identification of the first intermediate host, *A. balthica*. Furthermore, the phylogenetic analyses resulted in: (i) a re-identification/re-classification of 98 isolates including *D. baeri sensu* Galazzo *et al*. (2002); (ii) re-definition of the composition of the *D. baeri* species complex which now includes nine molecularly characterised species/lineages, i.e. *D. adamsi* (syn. *D. baeri sensu* Galazzo *et al*., 2002), *D. phoxini*, *Diplostomum* sp. Lineages 3–5 of Blasco-Costa *et al*. (2014), *Diplostomum* spp. 5–7 of Locke *et al*. (2015), and *Diplostomum* sp. of Lebedeva *et al*. (2021); (iii) re-definition of the composition of the *D. mergi* species complex which now includes seven molecularly characterised species/lineages, i.e. *D. parviventosum*, *D. mergi* Lineages 2 and 3 of Georgieva *et al*. (2013), *Diplostomum mergi* Lineage 4 of Selbach *et al*. (2015), *Diplostomum* spp. A and B of Kudlai *et al*. (2017), and *Diplostomum* sp. of Komatsu *et al*. (2019); and (iv) an updated nomenclature for the molecularly characterised species-level lineages of *Diplostomum*.

### Prevalence and life-cycle of *Diplostomum phoxini*

The summarised data for the prevalence of the molecularly characterised species/lineages of *Diplostomum* in the snail intermediate hosts in Europe (Table 6) indicate that prevalence is generally low when estimated from pooled samples (0.7–4.7%), whereas the prevalence estimated from distinct individual samples (i.e. as per the definition of Bush *et al*., 1997) ranges between 1.0 and 13.6% but is typically greater than 3.0%. The prevalence of *D. phoxini* in *A. balthica* studied in the River Ruhr fell within the latter range but with values greater than 3.0% in all distinct samples and a maximum prevalence of 13.6% recorded to date for *Diplostomum* spp. in Europe (Table 6; see also Online Resource Table S2 for details).

**Table 6.** Comparative data for the prevalence of *D. phoxini* and molecularly characterised species/lineages of *Diplostomum* spp. in intermediate snail hosts examined in Europe

Species	Host	Prevalence (%)	Locality	Source
*D. phoxini*	*Ampullaceana balthica*	3.3–13.6	River Ruhr, Germany	Present study
	*Radix auricularia* (as *Lymnaea auricularia*)	4.0–5.0	River Veude, France	Arvy and Buttner (1954)
	*Peregriana peregra* (as *Lymnaea pereger*)	3.9	Lake Fron Goch, UK	Rees (1957)
	*Ampullaceana balthica* (as *Lymnaea peregra ovata*)	0.9^a^	River Nagold, Germany	Dönges (1969*a*, 1969*b*)
	*Peregriana peregra* (as *Lymnaea peregra*)	3.4	Lake Fron Goch, UK	Bibby and Rees (1971)
*Diplostomum* sp. Lineage 2	*Ampullaceana balthica* (as *Radix peregra*)	0.7^b^	Lake Raudavatn, Iceland	Faltýnková *et al*. (2014)
*Diplostomum* sp. Lineage 4	*Ampullaceana balthica* (as *Radix peregra*)	2.8^b^	Lake Nordic House, Iceland	Faltýnková *et al*. (2014)
*Diplostomum* sp. Lineage 6	*Ampullaceana balthica* (as *Radix peregra*)	4.7^b^	Lake Nordic House, Iceland	Faltýnková *et al*. (2014)
*D. parviventosum*	*Radix auricularia*	3.1–7.1	Hengsteysee, Germany^c^	Selbach *et al.* (2015)
	*Ampullaceana lagotis* (as *Radix lagotis*)	0.4–1.5	Most Lake, Czech Republic	Vyhlídalová and Soldánová (2020)
*D. mergi* Lineage 2	*Radix auricularia*	2.1–6.7	Hengsteysee, Germany^c^	Selbach *et al.* (2015)
*D. mergi* Lineage 2	*Radix auricularia*	2.2–10.7	Sorpetalsperre, Germany^c^	Selbach *et al.* (2015)
*D. mergi* Lineage 3	*Radix auricularia*	1.0–3.1	Hengsteysee, Germany^c^	Selbach *et al.* (2015)
*D. mergi* Lineage 4	*Radix auricularia*	1.0	Hengsteysee, Germany^c^	Selbach *et al.* (2015)
*D. mergi* species complex	*Ampullaceana lagotis* (as *Radix lagotis*)	0.4–9.6^d^	Most Lake, Czech Republic	Vyhlídalová and Soldánová (2020)
*D. spathaceum*	*Radix auricularia*	2.1–4.1	Hengsteysee, Germany	Selbach *et al.* (2015)
	*Ampullaceana lagotis* (as *Radix lagotis*)	3.0–4.2	Most Lake, Czech Republic	Vyhlídalová and Soldánová (2020)
*Diplostomum* sp. Clade Q	*Radix auricularia*	3.6	Hengsteysee, Germany^c^	Selbach *et al.* (2015)

a Overall prevalence for pooled samples taken during 1959–1965.

b Data from pooled samples.

c Water reservoirs of the River Ruhr catchment area in North Rhine-Westphalia.

d Pooled data for all lineages of the *D. mergi* species complex.

Although most of the populations of *Diplostomum* spp. originate from lentic aquatic habitats (lakes, reservoirs, ponds) it is worth noting that both studies with prevalence estimated for multiple distinct samples originating from lotic waterbodies (River Ruhr in Germany, present study; River Veude in France, see Arvy and Buttner, 1954) revealed a high prevalence range of *D. phoxini* in both *A. balthica* and *R. auricularia*. This is in contrast with the expectation that flow conditions in the aquatic habitat affect digenean dispersal (Radke *et al*., 1961) with lentic habitats guaranteeing accelerated transmission rates (Soldánová and Kostadinova, 2011).

The data from our longitudinal study of the prevalence in *A. balthica* indicate that significant and consistent foci of infection with *D. phoxini* exist in the River Ruhr. This is further strengthened by the narrow transmission window estimated for *D. phoxini* in the riverine habitats studied. In Germany, the life span of *A. balthica* is estimated as one year, with copulation and egg-laying occurring in March (Glöer, 2002). We assume that in the River Ruhr juveniles hatch in April and the first patent infections in the new generation develop during June. This is supported by the fact that although large samples of snails were examined at all sites in May and June, the first patent infections with *D. phoxini* were registered as early as July. Our data thus indicate a transmission window of six months (June to November) with infection restart in each new snail generation.

Metacercariae of *D. phoxini* can survive in a minnow brain for up to five years and are accumulated by their fish hosts (Dönges, 1969*b*). This explains the maximum prevalence of 100% and high abundance of *D. phoxini* recorded in *P. phoxinus* host as reported previously (e.g. Arvy and Buttner, 1954; Rees, 1955, 1957). The longevity and accumulation of metacercariae in fish counteract the narrow transmission window for the larval stages and ensure the existence of a reservoir for maintenance of the infection with this species in the River Ruhr. However, the definitive host of *D. phoxini* is still poorly known since the only natural infection has been reported in *Mergus merganser* L. (the host of *D. pelmatoides* (Dubois, 1932), a synonym of *D. phoxini*). The distribution of this bird species in Germany is generally confined to the wintering areas around the coasts of the Baltic Sea (between October−November and March−April) and there is an isolated declining breeding population in Bavaria (Keller, 2009).

Recent observations, however, suggest an increase in the number of breeding pairs of *M. merganser* along the River Ruhr, that might contribute to the infection foci in *A. balthica* along the river.

Shigin (1986, 1993) reported the existence of intense and persistent foci of infection with *D. phoxini* in fish populations of waterbodies where *Mergus* spp. are practically lacking. Studies on the life-cycle indicate that *D. phoxini* is not highly specific to its definitive host as adult flukes containing eggs and mature sperm have been obtained from both avian and mammalian hosts. Adults of *D. phoxini* have been raised experimentally in ducklings of *Anas platyrhynchos* (see Arvy and Buttner, 1954; Dönges, 1969*a*, 1969*b*; Erasmus, 1969), *Cairina moschata domestica* (see Arvy and Buttner, 1954), *L. argentatus* (see Berrie, 1960) and laboratory mice (Berrie, 1960; Shigin, 1986, 1993), but not in *L. ridibundus* (see Dönges, 1969*a*); a very rapid rate of development in ducklings (3–5 days post-infection) has been observed (Rees, 1955; Berrie, 1960; Dönges, 1969*a*, 1969*b*; Erasmus, 1969). This rapid rate of adult development and compatibility with anatid and mammalian hosts, in association with a possibility of parasite-induced changes in fish behaviour and mortality at high intensity of infection levels, tend to support the hypothesis that purely facultative ichthyophages such as aquatic rodents (Shigin, 1986, 1993) and/or *A. platyrhynchos* (see Miroshnichenko and Sten'ko, 1983) may also act as definitive hosts of *D. phoxini*.

### *Diplostomum baeri* species complex

Including in the phylogenetic analysis *D. phoxini* and the additional 26 species-level lineages molecularly characterised during 2015–2021 resulted in a change of the composition of the *D. baeri* species complex with the inclusion of *D. phoxini* and *Diplostomum* sp. of Lebedeva *et al*. (2021) and the exclusion of *Diplostomum* sp. 2 of Moszczynska *et al*. (2009) which was associated with three North American lineages (*Diplostomum* spp. 12, 18 and 19 of Locke *et al*., 2015) sequenced recently by Locke *et al*. (2015).

Metacercariae of all species/lineages of the *D. baeri* species complex represent non-lens-dwelling forms recovered from the eye vitreous humour and retina and the brain of the fish hosts. The microhabitat within the fish outside the lens utilised by the metacercariae of *Diplostomum* spp. is an important species characteristic (Shigin, 1986) and defining the exact location of the metacercariae can facilitate identification/differentiation as illustrated by Blasco-Costa *et al*. (2014) who sequenced and differentiated morphologically two species-level lineages (*Diplostomum* sp. Lineage 3 from the vitreous humour of the eye and *Diplostomum* sp. Lineage 5 from the eye retina) in the salmonids *Salmo trutta* L. and *Salvelinus alpinus* (L.) and two lineages (*Diplostomum* sp. Lineage 4 from the eye retina and brain and *Diplostomum* sp. Lineage 6 from the retina) in the gasterosteid *Gasterosteus aculeatus* L.

Unfortunately, Locke *et al*. (2010*a*) made no distinction between the sub-retinal space, retina and vitreous humour of the eye “because metacercariae in these sites often detach in frozen material”. This applies to six species/lineages for which additional clarification of the metacercarial microhabitat in fish is required based on examination of unfrozen material: *D. baeri sensu* Galazzo *et al*. (2002); *Diplostomum* sp. 2 of Moszczynska *et al*. (2009); and *Diplostomum* spp. 5–9 of Locke *et al*. (2010*a*). Locke *et al*. (2015) applied the division of “lens” *vs* “non-lens (eye)” for these species and for five additional lineages (*Diplostomum* spp. 12, 13, 17–19). Overall, there is conflicting information for the location of the metacercariae between the two large inventories of Locke *et al*. (2010*a*) and Locke *et al*. (2015) and between the text and the supplementary data of Locke *et al*. (2015) for 12 isolates of one lineage (*Diplostomum* sp. 2 of Moszczynska *et al*. (2009)) and nine isolates of six lineages, respectively (highlighted in red in Online Resource Table S5). Regarding the lineages of the *D. baeri* species complex, conflicting information for isolate microhabitats in the fish hosts has been provided for one isolate (KR271039) of *D. baeri sensu* Galazzo *et al*. (2002) and five isolates of *Diplostomum* sp. 7 of Locke *et al*. (2010*a*) (KR271398, KR271399, KR271402, KR271404, KR271407) (see Online Resource Table S5; Locke *et al*., 2015).

### What is *D. baeri* sensu Galazzo *et al*. (2002)?

In the *Guide to the Parasites of Fishes of Canada*, Gibson (1996) provided a key for the metacercariae of seven species of *Diplostomum*, including three non-lens-dwelling forms: *D. scudderi* (Olivier, 1941) Dubois, 1966 (syn. *Diplostomulum baeri eucaliae* Hoffman & Hundley, 1957) from the brain or retina of gasterosteids; *D. baeri bucculentum* Dubois & Rausch, 1948 from the retina or vitreous humour of the eye of salmonids; and *D. adamsi* Lester & Huizinga, (1977) from the retina of *Perca flavescens* (Mitchill).

Galazzo *et al*. (2002) developed experimentally adults in *Larus delawarensis* fed metacercariae from the “vitreous humour” of *P. flavescens* collected in the St Lawrence River near Montreal, Canada. These authors found a substantial differentiation (3.8%, 23 nt positions) in the ITS1 rDNA region between the specimens sequenced in North America and Europe and concluded that the two forms are not conspecific. However, Galazzo *et al*. (2002) used the name *D. baeri* for their experimentally developed adults. Locke *et al*. (2010*a*) generated *cox*1 sequences “from archived DNA of three vouchered adult specimens” studied by Galazzo *et al*. (2002) and from eight additional adult specimens and 64 metacercariae from the “vitreous humour” of *P. flavescens* collected in Canada. These authors also used the name *D. baeri* based on the sequence matching with the adults identified by Galazzo *et al*. (2002).

All recent molecular phylogenies indicate that the North American lineage named as *D. baeri* by Galazzo *et al*. (2002) and Locke *et al*. (2010*a*, 2010*b*, 2015) and the species-level lineages of the *D. baeri* complex are genetically distinct (Georgieva *et al*., 2013; Blasco-Costa *et al*., 2014; Faltýnková *et al*., 2014; Selbach *et al*., 2015; Soldánová *et al*., 2017) and the present analyses strongly support this (Figs 5, 6; Table 4; Online Resource Fig. S2). Therefore, there is no justification for perpetuating use of this name for the North American lineage from *P. flavescens* and *Larus* spp. Compared with the original description of *D. baeri* based on specimens from Europe, the experimentally obtained material measured and illustrated by Galazzo *et al*. (2002) differs in having: a much larger body with a longer and narrower forebody and a substantially longer and narrower hindbody; an oral sucker much larger than pharynx (mean OSW/PHW = 1.5 *vs* oral sucker slightly larger than pharynx; OSW/PHW = 0.96–1.26) that is also equal to ventral sucker (mean VSW/OSW = 1.01 *vs* oral sucker slightly smaller than ventral sucker in *D. baeri*). Additionally, the anterior margins of the vitelline fields reach to the level of ventral sucker in the material described by Galazzo *et al*. (2002) whereas they extend anteriorly to ventral sucker up to mid-distance between pharynx and ventral sucker in *D. baeri* (see Dubois, 1970).

Unfortunately, Galazzo *et al*. (2002) did not compare their material with the description of *D. adamsi*, the only species with metacercariae known to develop in *P. flavescens* in Canada and North America in general (see Gibson, 1996; Zelmer and Arai, 1998). The life-cycle of *D. adamsi* was completed experimentally by Lester and Huizinga (1977) using *Lymnaea stagnalis* (L.) and *Ladislavella elodes* (Say) as the first intermediate hosts, *P. flavescens* as the only susceptible host out of five fish species tested, and *Larus argentatus* Pontoppidan as the experimental definitive host. In addition to the detailed descriptions of the life-cycle stages of *D. adamsi*, these authors provided histological and scanning electron microscopy evidence for the microhabitat of the metacercariae in *P. flavescens*, i.e. “in the peripheral retina, in a cavity between the photoreceptor cells and the pigment epithelium”.

The solution for the confusion with the identification of *D. baeri sensu* Galazzo *et al*. (2002) comes from the detailed histological study of Ubels *et al*. (2018) clearly showing that infection with *D. baeri sensu* Galazzo *et al*. (2002) is confined to tissues associated with the eye retina (choroidal vasculature) of *P. flavescens*; these authors also generated sequence data for the metacercariae from the retinal tissues of *P. flavescens*. As shown in Online Resource Fig. S2, these sequences clustered with the sequences from the same fish host and *Larus* spp. in the studies of Galazzo *et al*. (2002), Moszczynska *et al*. (2009) and Locke *et al*. (2010*a*, 2010*b*, 2015). All of the above considerations clearly suggest that the metacercariae originating from *P. flavescens* and sequenced by these authors and by Ubels *et al*. (2018) represent the retinal form *D. adamsi*. The reclassification of the sequences labelled as “*Diplostomum baeri* complex sp. LIN2” and “*Diplostomum* aff. *baeri* LIN2” by Gordy and Hanington (2019) (see above) provides molecular evidence that, in agreement with the original description of *D. adamsi*, the snail *L. elodes* acts as the first intermediate host of this species. The introduction of *D. adamsi* as the only plausible identification for the lineage *D. baeri sensu* Galazzo *et al*. (2002) sequenced by Galazzo *et al*. (2002), Locke *et al*. (2010*a*, 2015) and Gordy and Hanington (2019) does not require changing the name of the *D. baeri* species complex as there is a number of lineages within it awaiting taxonomic scrutiny.

### Re-classification and an updated nomenclature for *Diplostomum* spp.

Based on the present phylogenetic analyses, an updated nomenclature was applied and a large number of isolates of *Diplostomum* spp. published before 29 June 2021 was re-identified/re-classified (Table 4, Fig. 5, Online Resource Table S5, Fig. S2). The present re-classification revealed new linkages between life-cycle stages for three species/lineages, i.e. *D. adamsi*, *Diplostomum* sp. 13 of Locke *et al*. (2015) and *Diplostomum* sp. 18 of Locke *et al*. (2015). Cercarial isolates of these forms were sequenced from *L. elodes* in Canada by Gordy and Hanington (2019) (see Table 4 and Online Resource Table S5).

Here, we would like to highlight two cases with relevance to the data for the *D. baeri* complex discussed above. Landeryou *et al*. (2020) used *cox*1 and ITS sequences to identify the metacercariae from the vitreous humour of *Salmo trutta* collected in Scotland and used for characterisation of the mitochondrial genome of a species they believed to be *D. baeri*. However, these authors selected for their analysis *cox*1 sequences for just two lineages of the *D. baeri* species complex, i.e. *D. baeri sensu* Galazzo *et al*. (2002) from North America and *Diplostomum* sp. Lineage 3 of Blasco-Costa *et al*. (2014) (the “trout clade” of the *D. baeri* species complex *sensu* Georgieva *et al*., 2013). Although the *cox*1 sequences of Landeryou *et al*. (2020) clearly fell within the clade of *Diplostomum* sp. Lineage 3 of Blasco-Costa *et al*. (2014) (the “trout clade” of Georgieva *et al*., 2013), they named the species as “*D. baeri*”. Our analysis revealed that the material sequenced by Landeryou *et al*. (2020) in fact belongs to and should be referred to as *Diplostomum* sp. Lineage 3 of Blasco-Costa *et al*. (2014) (Table 4, Online Resource Table S5 and Fig. S2).

Ubels *et al*. (2018) reported as *D. baeri* two sequences from metacercariae ex *P. flavescens* and *Luxilus cornutus* (Mitchill) collected in Douglas Lake, Michigan, USA. However, there is a conflict with host annotation in their paper and the Supplementary Fig. S1 provided by these authors (see Table 4). Whichever the host, our analysis clearly showed that the sequence MF142178 belongs to *Diplostomum* sp. 3 of Moszczynska *et al*. (2009) and the sequence MF142161 belongs to *Diplostomum* sp. 4 of Moszczynska *et al*. (2009).

The nomenclature of the genetic lineages of *Diplostomum* is in a state of flux since scientific names for 35 species-level lineages of *Diplostomum* have not yet been suggested. Identification to the species level *via* linking the genetic and morphological data for these lineages will be a long process and some lineages will remain unidentified for indefinite time. Locke *et al*. (2015) highlighted the problems associated with name discrepancies in the publications *vs* GenBank annotations for the expanding number of molecularly delineated species-level lineages within the Diplostomidae. Whilst we agree with their criticisms, we should like to highlight that the publication should be the leading source for the identification and host/microhabitat data for the newly sequenced isolates and the precise linking to GenBank sequences (and their annotations) should be part of the publication. In an ideal world with a centralised system for registering the lineage number sequence, the numbering system would be effective (as suggested by Locke *et al*., 2015) but this is not the case; the same applies to a lettering system, e.g. there are pairs of lineages currently labelled as A, B and C (see Kudlai *et al*., 2017; Gordy and Hanington, 2019).

Lineage ‘names’ (labels) are not species binomens and thus no compliance with the International Code of Zoological Nomenclature is required. However, it would be wise to follow Code's principles of homonymy and priority of publication to ensure that the ‘name’ of each genetic lineage is unique and distinct, and that the oldest available ‘name’ is used for already characterised lineages. The uniqueness is ensured by consistently using the ‘name’ in association with the reference of the first publication, e.g. *Diplostomum* sp. 1 of Moszczynska *et al*. (2009), *Diplostomum* sp. A of Gordy and Hanington (2019), *Diplostomum* sp. A of Kudlai *et al*. (2017), *Diplostomum* sp. Lineage 2 of Blasco-Costa *et al*. (2014) or simply *Diplostomum* sp. of Chibwana *et al*. (2013) (see Table 4 for all updated lineage labels).

The updated data on the nomenclature and distribution for molecularly characterised species/lineages of *Diplostomum* based on our global analysis provided in Table 4 indicate that, in spite of the accumulation of sequences from recent studies, current distribution of *Diplostomum* spp. is the result of uneven sampling effort and suggest our knowledge of the species and genetic diversity in this group is still rudimentary in Africa, Asia and South America. Thus, nearly half of the molecularly characterised species/lineages (21; 48%) have only been recorded in North America. Of these, 12 (57%) taxa, including four singletons, have only been recorded in Canada. Nearly a third of the species/lineages (12 taxa, 27%, including 3 singletons) have only been recorded in Europe and there are fewer molecular records from Asia (eight taxa, including three also found in Europe: *D. spathaceum*; *D. mergi* Lineage 2 of Georgieva *et al*. (2013); *D. mergi* Lineage 4 of Selbach *et al*. (2015)) and Africa (three taxa, including two also found in Asia: *Diplostomum* spp. 14 and 16 of Locke *et al*. (2015)). Just one species has been characterised molecularly in South America.

Finally, the present updated synopsis of *Diplostomum* species/lineages highlights an important caveat for enhancing the knowledge of the diversity of *Diplostomum* spp. in fish hosts, i.e. the virtual lack of sequences for metacercariae of salmonid and gasterosteid hosts from North America. Currently, only four sequences are available from these host groups, three sequences from metacercariae in salmonids, two for *Diplostomum* sp. 7 and one for *Diplostomum* sp. 9, and a single sequence for *Diplostomum* sp. 13 of Locke *et al*. (2015) (possibly *D. scudderi* (Olivier, 1941)) from *G. aculeatus*. We predict that, similar to the current situation in Europe, focused sampling of gasterosteids and salmonids with a careful identification of the location of the non-lens-dwelling metacercariae will reveal a number of additional species/lineages of the *D. baeri* complex in North America. Furthermore, precise identification of the microhabitat in salmonid hosts anchored to novel morphological and sequence data may help assess the status of *D. baeri bucculentum* Dubois & Rausch, 1948 and distinguish it from the retinal form reported from salmonoids in Canada (see Gibson, 1996) and from the European lineages molecularly and morphologically characterised by Blasco-Costa *et al*. (2014) and described by Faltýnková *et al*. (2014). Sequencing of metacercariae from salmonids and gasterosteids will also provide additional data for testing the hypothesis for North America being an ancestral area for the *D. baeri* species complex (Blasco-Costa *et al*., 2014) and shed light on the evolution of this group.

## Data Availability

The data supporting the findings of this study are available within the article and its supplementary materials. All newly generated sequences were deposited in the GenBank database under the following accession numbers: MZ615631-MZ615639 (*cox*1, *D. phoxini*); MZ616379 and MZ616380 (28S, *D. phoxini*); MZ616381 and MZ616382 (ITS1-5.8S-ITS2, *D. phoxini*); MZ615629 and MZ615630 (*cox*1, *A. balthica*); MZ616383 (28S, *A. balthica*); and MZ616378 (ITS2, *A. balthica*). Raw data are available on request from the corresponding author [JS].

## Supplementary material

For supplementary material accompanying this paper visit https://doi.org/10.1017/S0031182021001372. click here to view supplementary material

## References

[ref1] Aksenova OV, Bolotov IN, Gofarov MY, Kondakov AV, Vinarski MV, Bespalaya YV, Kolosova YS, Palatov DM, Sokolova SE, Spitsyn VM, Tomilova AA, Travina OV and Vikhrev IV (2018) Species richness, molecular taxonomy and biogeography of the radicine pond snails (Gastropoda: Lymnaeidae) in the Old World. Scientific Reports 8, 11199. 30046044 10.1038/s41598-018-29451-1 PMC6060155

[ref2] Anderson MJ, Gorley RN and Clarke KR (2008) PERMANOVA+for PRIMER: Guide to Software and Statistical Methods. Plymouth, UK: PRIMER-E.

[ref3] Arvy L and Buttner A (1954) Données sur le cycle évolutif de *Diplostomulum phoxini* (Faust, 1918) (Trematoda, Diplostomidae). Comptes Rendus Hebdomadaires Séances de l´Académie des Sciences Paris Serie C 239, 1085–1087.

[ref4] Behrmann-Godel J (2013) Parasite identification, succession and infection pathways in perch fry (*Perca fluviatilis*): new insights through a combined morphological and genetic approach. Parasitology 140, 509–520. 23279837 10.1017/S0031182012001989

[ref5] Berrie AD (1960) The influence of various definitive hosts on the development of *Diplostomum phoxini* (Strigeida, Trematoda). Journal of Helminthology 34, 205–210.

[ref6] Bibby MC and Rees G (1971) The ultrastructure of the epidermis and associated structures in the metacercaria cercaria and sporocyst of *Diplostomum phoxini* (Faust, 1918). Zeitschrift für Parasitenkunde 37, 169–186. 5098549 10.1007/BF00259497

[ref7] Blasco-Costa I, Faltýnková A, Georgieva S, Skírnisson K, Scholz T and Kostadinova A (2014) Fish pathogens near the Arctic circle: molecular, morphological and ecological evidence for unexpected diversity of *Diplostomum* (Digenea: Diplostomidae) in Iceland. International Journal for Parasitology 44, 703–715. 24929135 10.1016/j.ijpara.2014.04.009

[ref8] Brabec J, Kostadinova A, Scholz T and Littlewood DTJ (2015) Complete mitochondrial genomes and nuclear ribosomal RNA operons of two species of *Diplostomum* (Platyhelminthes: Trematoda): a molecular resource for taxonomy and molecular epidemiology of important fish pathogens. Parasites & Vectors 8, 336. 26084797 10.1186/s13071-015-0949-4 PMC4477422

[ref9] Bush AO, Lafferty KD, Lotz JM and Shostak AW (1997) Parasitology meets ecology on its own terms: Margolis revisited. Journal of Parasitology 83, 575. 9267395

[ref10] Chibwana FD, Blasco-Costa I, Georgieva S, Hosea KM, Nkwengulila G, Scholz T and Kostadinova A (2013) A first insight into the barcodes for African diplostomids (Digenea: Diplostomidae): brain parasites in *Clarias gariepinus* (Siluriformes: Clariidae). Infection, Genetics and Evolution 17, 62–70. 10.1016/j.meegid.2013.03.037 23542455

[ref11] Darriba D, Taboada GL, Doallo R and Posada D (2012) JModelTest 2: more models, new heuristics and parallel computing. Nature Methods 9, 772. 10.1038/nmeth.2109 PMC4594756 22847109

[ref12] Dönges J (1969 *a*) *Diplostomum phoxini* (Faust, 1918) (Trematoda). Morphologie des Miracidiums sowie Beobachtungen an weiteren Entwicklungsstadien. Zeitschrift für Parasitenkunde 32, 120–127. 5794767 10.1007/BF00259974

[ref13] Dönges J (1969 *b*) Entwicklungs- und Lebensdauer von Metacercarien. Zeitschrift für Parasitenkunde 31, 340–366. 5379822 10.1007/BF00259732

[ref100] Dubois G (1970) Synopsis des Strigeidae et des Diplostomatidae (Trematoda). Mémoires de la Société Neuchateloise des Sciences Naturelles 10, 259–727.

[ref14] Enabulele EE, Awharitoma AO, Lawton SP and Kirk RS (2018) First molecular identification of an agent of diplostomiasis, *Diplostomum pseudospathaceum* (Niewiadomska 1984) in the United Kingdom and its genetic relationship with populations in Europe. Acta Parasitologica 63, 444–453. 29975660 10.1515/ap-2018-0054

[ref15] Erasmus DA (1969) Studies on the host-parasite interface of strigeid trematodes. VI. Ultrastructural observations on the lappets of *Diplostomum phoxini* Faust, 1918. Zeitschrift für Parasitenkunde 32, 48–58. 5793650 10.1007/BF00259961

[ref16] Faltýnková A, Georgieva S, Kostadinova A, Blasco-Costa I, Scholz T and Skírnisson K (2014) *Diplostomum* von Nordmann, 1832 (Digenea: Diplostomidae) in the sub-Arctic: descriptions of the larval stages of six species discovered recently in Iceland. Systematic Parasitology 89, 195–213. 25301510 10.1007/s11230-014-9517-0

[ref17] Galazzo DE, Dayanandan S, Marcogliese DJ and McLaughlin JD (2002) Molecular systematics of some North American species of *Diplostomum* (Digenea) based on rDNA-sequence data and comparisons with European congeners. Canadian Journal of Zoology 80, 2207–2217.

[ref18] Georgieva S, Soldánová M, Pérez-del-Olmo A, Dangel DR, Sitko J, Sures B and Kostadinova A (2013) Molecular prospecting for European *Diplostomum* (Digenea: Diplostomidae) reveals cryptic diversity. International Journal for Parasitology 43, 57–72. 23201234 10.1016/j.ijpara.2012.10.019

[ref19] Gibson DI (1996) Guide to the Parasites of Fishes of Canada. Part IV. Trematodes. Ottawa, Canada: NRC Press.

[ref20] Glöer P (2002) Die Süßwassergastropoden Nord- und Mitteleuropas – Bestimmungsschlüssel, Lebensweise, Verbreitung. Hackenheim, Germany: Conch Books.

[ref21] Gordy MA and Hanington PC (2019) A fine-scale phylogenetic assessment of digenean trematodes in central Alberta reveals we have yet to uncover their total diversity. Ecology and Evolution 9, 3153–3238. 30962888 10.1002/ece3.4939 PMC6434566

[ref22] Gordy MA, Kish L, Tarrabain M and Hanington PC (2016) A comprehensive survey of larval digenean trematodes and their snail hosts in central Alberta, Canada. Parasitology Research 115, 3867–3880. 27245072 10.1007/s00436-016-5152-9

[ref23] Hoogendoorn C, Smit NJ and Kudlai O (2020) Resolution of the identity of three species of *Diplostomum* (Digenea: Diplostomidae) parasitising freshwater fishes in South Africa, combining molecular and morphological evidence. International Journal for Parasitology: Parasites and Wildlife 11, 50–61. 31908920 10.1016/j.ijppaw.2019.12.003 PMC6938850

[ref24] Huelsenbeck JP, Ronquist F, Nielsen R and Bollback JP (2001) Bayesian inference of phylogeny and its impact on evolutionary biology. Science (New York, N.Y.) 294, 2310–2314. 10.1126/science.1065889 11743192

[ref25] Katoh K, Rozewicki J and Yamada KD (2019) MAFFT online service: multiple sequence alignment, interactive sequence choice and visualization. Briefings in Bioinformatics 20, 1160–1166. 28968734 10.1093/bib/bbx108 PMC6781576

[ref26] Keller V (2009) The goosander *Mergus merganser* population breeding in the Alps and its connections to the rest of Europe. Wildfowl Special Issue 2, 60–73.

[ref27] Komatsu N, Itoh N and Ogawa K (2019) Worm cataract of hatchery-reared Japanese dace *Tribolodon hakonensis* caused by *Diplostomum* sp. (Digenea: Diplostomidae). Fish Pathology 54, 1–11.

[ref28] Kottelat M and Freyhof J (2007) Handbook of European Freshwater Fishes. Berlin: Publications Kottelat, Cornol and Freyhof, 646 pp.

[ref29] Kudlai O, Oros M, Kostadinova A and Georgieva S (2017) Exploring the diversity of *Diplostomum* (Digenea: Diplostomidae) in fishes from the River Danube using mitochondrial DNA barcodes. Parasites & Vectors 10, 592. 29197405 10.1186/s13071-017-2518-5 PMC5712130

[ref30] Kuhn JA, Kristoffersen R, Knudsen R, Jakobsen J, Marcogliese DJ, Locke SA, Primicerio R and Amundsen PA (2015) Parasite communities of two three-spined stickleback populations in subarctic Norway – effects of a small spatial-scale host introduction. Parasitology Research 114, 1327–1339. 25630694 10.1007/s00436-015-4309-2

[ref31] Kumar S, Stecher G and Tamura K (2016) MEGA7: molecular evolutionary genetics analysis version 7.0 for bigger datasets. Molecular Biology and Evolution 33, 1870–1874. 27004904 10.1093/molbev/msw054 PMC8210823

[ref32] Kuraku S, Zmasek CM, Nishimura O and Katoh K (2013) ALeaves facilitates on-demand exploration of metazoan gene family trees on MAFFT sequence alignment server with enhanced interactivity. Nucleic Acids Research 41, 22–28. 10.1093/nar/gkt389 PMC3692103 23677614

[ref33] Landeryou T, Kett SM, Ropiquet A, Wildeboer D and Lawton SP (2020) characterisation of the complete mitochondrial genome of *Diplostomum baeri*. Parasitology International 79, 102166. 32603866 10.1016/j.parint.2020.102166

[ref34] Lebedeva DI, Chrisanfova GG, Ieshko EP, Guliaev AS, Yakovleva GA, Mendsaikhan B and Semyenova SK (2021) Morphological and molecular differentiation of *Diplostomum* spp. metacercariae from brain of minnows (*Phoxinus phoxinus* L.) in four populations of Northern Europe and East Asia. Infection Genetics & Evolution **92**, 104911. doi: 10.1016/j.meegid.2021.104911 33991672

[ref35] Lester RJ and Huizinga HW (1977) *Diplostomum adamsi* sp. n.: description, life cycle, and pathogenesis in the retina of *Perca flavescens*. Canadian Journal of Zoology 55, 64–73. 837282 10.1139/z77-007

[ref36] Locke SA, McLaughlin JD, Dayanandan S and Marcogliese DJ (2010 *a*) Diversity and specificity in *Diplostomum* spp. metacercariae in freshwater fishes revealed by cytochrome c oxidase I and internal transcribed spacer sequences. International Journal for Parasitology 40, 333–343. 19737570 10.1016/j.ijpara.2009.08.012

[ref37] Locke SA, McLaughlin JD and Marcogliese DJ (2010 *b*) DNA barcodes show cryptic diversity and a potential physiological basis for host specificity among Diplostomoidea (Platyhelminthes: Digenea) parasitizing freshwater fishes in the St. Lawrence River, Canada. Molecular Ecology 19, 2813–2827. 20561195 10.1111/j.1365-294X.2010.04713.x

[ref38] Locke SA, Al-Nasiri FS, Caffara M, Drago F, Kalbe M, Lapierre AR, McLaughlin JD, Nie P, Overstreet RM, Souza GTR, Takemoto RM and Marcogliese DJ (2015) Diversity, specificity and speciation in larval Diplostomidae (Platyhelminthes: Digenea) in the eyes of freshwater fish, as revealed by DNA barcodes. International Journal for Parasitology 45, 841–855. 26276524 10.1016/j.ijpara.2015.07.001

[ref39] Locke SA, Drago FB, Núñez V, Souza GTRE and Takemoto RM (2020) Phylogenetic position of *Diplostomum* spp. from New World herons based on complete mitogenomes, rDNA operons, and DNA barcodes, including a new species with partially elucidated life cycle. Parasitology Research 119, 2129–2137. 32472382 10.1007/s00436-020-06713-4

[ref40] Miller MA, Pfeiffer W and Schwartz T (2010) Creating the CIPRES Science Gateway for inference of large phylogenetic trees. In *2010 Gateway Computing Environments Workshop, GCE 2010, p. IEEE*.

[ref41] Minelli A and Fusco G (2010) Developmental plasticity and the evolution of animal complex life cycles. Philosophical Transactions of the Royal Society B: Biological Sciences 365, 631–640. 10.1098/rstb.2009.0268 PMC2817148 20083638

[ref42] Miroshnichenko AI and Sten'ko RP (1983) Effect of anthropogenic factors on a diplostomosis focus on Crimea. In *11th All-Union Congress of Parasitocenologists*. Kiev, Ukraine: Naukova Dumka, pp. 223–224.

[ref43] Moszczynska A, Locke SA, McLaughlin JD, Marcogliese DJ and Crease TJ (2009) Development of primers for the mitochondrial cytochrome c oxidase I gene in digenetic trematodes (Platyhelminthes) illustrates the challenge of barcoding parasitic helminths. Molecular Ecology Resources 9, 75–82. 21564967 10.1111/j.1755-0998.2009.02634.x

[ref44] Niewiadomska K and Kiselienė V (1994) *Diplostomum* cercariae (Digenea) in snails of Lithuania. II. Survey of species. Acta Parasitologica 31, 179–186.

[ref45] Olson PD, Cribb TH, Tkach VV, Bray RA and Littlewood DTJ (2003) Phylogeny and classification of the Digenea (Platyhelminthes: Trematoda). International Journal for Parasitology 33, 733–755. 12814653 10.1016/s0020-7519(03)00049-3

[ref46] Pérez-del-Olmo A, Georgieva S, Pula HJ and Kostadinova A (2014) Molecular and morphological evidence for three species of *Diplostomum* (Digenea: Diplostomidae), parasites of fishes and fish-eating birds in Spain. Parasites & Vectors 7, 502. 25388753 10.1186/s13071-014-0502-x PMC4242471

[ref47] Radke MG, Ritchie LS and Rowan WB (1961) Effects of water velocities on worm burdens of animals exposed to *Schistosoma mansoni* cercariae released under laboratory and field conditions. Experimental Parasitology 11, 323–331. 14489754 10.1016/0014-4894(61)90039-x

[ref48] Rahn AK, Krassmann J, Tsobanidis K, MacColl ADC and Bakker TCM (2016) Strong neutral genetic differentiation in a host, but not in its parasite. *Infection*. Genetics and Evolution 44, 261–271. 10.1016/j.meegid.2016.07.011 27421211

[ref49] Rees G (1955) The adult and diplostomulum stage (*Diplostomulum phoxini* (Faust) of *Diplostomum pelmatoides* Dubois and an experimental demonstration of part of the life cycle. Parasitology 45, 295–312. 13280271 10.1017/s0031182000027670

[ref50] Rees G (1957) *Cercaria Diplostomi phoxini* (Faust), a furcocercaria which develops into *Diplostomulum phoxini* in the brain of the minnow. Parasitology 47, 126–137.

[ref51] Ronquist F, Teslenko M, Van Der Mark P, Ayres DL, Darling A, Höhna S, Larget B, Liu L, Suchard MA and Huelsenbeck JP (2012) MrBayes 3.2: efficient Bayesian phylogenetic inference and model choice across a large model space. Systematic Biology 61, 539–542. 22357727 10.1093/sysbio/sys029 PMC3329765

[ref52] Rozas J, Sanchez-DelBarrio JC, Messeguer X and Rozas R (2003) DnaSP, DNA polymorphism analyses by the coalescent and other methods. Bioinformatics (Oxford, England) 19, 2496–2497. 10.1093/bioinformatics/btg359 14668244

[ref53] Rudko SP, Reimink RL, Froelich K, Gordy MA, Blankespoor CL and Hanington PC (2018) Use of qPCR-based cercariometry to assess swimmer’s itch in recreational lakes. EcoHealth 15, 827–839. 30120669 10.1007/s10393-018-1362-1 PMC6267424

[ref54] Schwelm J, Soldánová M, Vyhlídalová T, Sures B and Selbach C (2018) Small but diverse: larval trematode communities in the small freshwater planorbids *Gyraulus albus* and *Segmentina nitida* (Gastropoda: Pulmonata) from the Ruhr River, Germany. Parasitology Research 117, 241–255. 29222665 10.1007/s00436-017-5699-0

[ref55] Selbach C, Soldánová M, Georgieva S, Kostadinova A and Sures B (2015) Integrative taxonomic approach to the cryptic diversity of *Diplostomum* spp. in lymnaeid snails from Europe with a focus on the ‘*Diplostomum mergi*’ species complex. Parasites & Vectors 8, 300. 26036245 10.1186/s13071-015-0904-4 PMC4476078

[ref56] Shigin AA (1986) [Trematode fauna of the USSR. Genus Diplostomum. Metacercariae]. Moscow, Russia: Nauka (in Russian).

[ref57] Shigin AA (1993) [Trematodes of the Fauna of Russia and Neighbouring Regions. Genus Diplostomum. Adults]. Moscow, Russia: Nauka (in Russian).

[ref58] Soldánová M and Kostadinova A (2011) Rapid colonisation of *Lymnaea stagnalis* by larval trematodes in eutrophic ponds in central Europe. International Journal for Parasitology 41, 981–990. 21712042 10.1016/j.ijpara.2011.05.005

[ref59] Soldánová M, Georgieva S, Roháčová J, Knudsen R, Kuhn JA, Henriksen EH, Siwertsson A, Shaw JC, Kuris AM, Amundsen PA, Scholz T, Lafferty KD and Kostadinova A (2017) Molecular analyses reveal high species diversity of trematodes in a sub-Arctic lake. International Journal for Parasitology 47, 327–345. 28315362 10.1016/j.ijpara.2016.12.008

[ref60] Sonnenberg R, Nolte A and Tautz D (2007) An evaluation of LSU rDNA D1–D2 sequences for their use in species identification. Frontiers in Zoology 4, 6. 17306026 10.1186/1742-9994-4-6 PMC1805435

[ref61] Telford MJ, Herniou EA, Russell RB and Littlewood DTJ (2000) Changes in mitochondrial genetic codes as phylogenetic characters: two examples from the flatworms. Proceedings of the National Academy of Sciences of the United States of America 97, 11359–11364. 11027335 10.1073/pnas.97.21.11359 PMC17205

[ref62] Ubels JL, DeJong RJ, Hoolsema B, Wurzberger A, Nguyen TT, Blankespoor HD and Blankespoor CL (2018) Impairment of retinal function in yellow perch (*Perca flavescens*) by *Diplostomum baeri* metacercariae. International Journal for Parasitology: Parasites and Wildlife 7, 171–179. 29988865 10.1016/j.ijppaw.2018.05.001 PMC6032499

[ref63] Van Steenkiste N, Locke SA, Castelin M, Marcogliese DJ and Abbott CL (2015) New primers for DNA barcoding of digeneans and cestodes (Platyhelminthes). Molecular Ecology Resources 15, 945–952. 25490869 10.1111/1755-0998.12358

[ref64] Vyhlídalová T and Soldánová M (2020) Species-specific patterns in cercarial emergence of *Diplostomum* spp. from snails *Radix lagotis*. International Journal for Parasitology 50, 1177–1188. 32896571 10.1016/j.ijpara.2020.07.009

[ref65] Zelmer DA and Arai HP (1998) The contributions of host age and size to the aggregated distribution of parasites in yellow perch, *Perca flavescens*, from Garner Lake, Alberta, Canada. Journal of Parasitology 84, 24–28. 9488333

