# Supplementary material for: Molecular and morphological characterisation of Diplostomum phoxini (Faust, 1918) with a revised classification and an updated nomenclature of the species-level lineages of Diplostomum (Digenea: Diplostomidae) sequenced worldwide
Source: Parasitology. 2021 Aug 9;148(13):1648–64. doi: 10.1017/S0031182021001372 (PMC8564804; doi:10.1017/S0031182021001372)

Online Resource Fig. S1 Phylogram from neighbour-joining (NJ) analysis of the *cox*1 sequence alignment (42 sequences; 636 nt) for 36 representatives of the subfamily Amphipepleinae (Mollusca: Lymnaeidae). Outgroup: *Galba truncatula*. Bootstrap support values ≥ 70 are provided above branches. The scale-bar indicates the expected number of substitutions per site

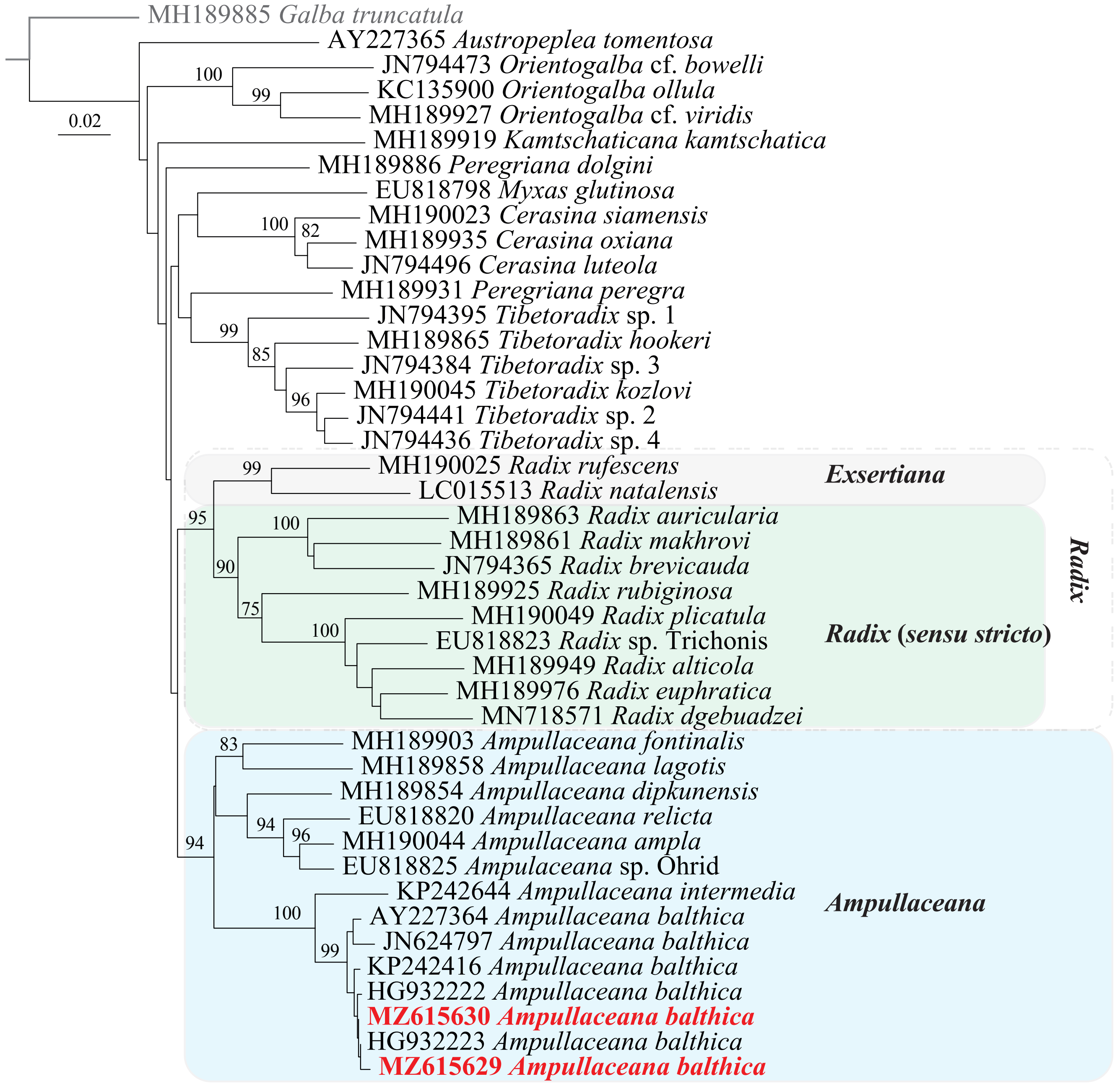

Supplement: Supplementary file 1 [file S0031182021001372sup001.zip › S0031182021001372sup001/S0031182021001372sup001.pdf]
