# Supplementary material for: Molecular and morphological characterisation of Diplostomum phoxini (Faust, 1918) with a revised classification and an updated nomenclature of the species-level lineages of Diplostomum (Digenea: Diplostomidae) sequenced worldwide
Source: Parasitology. 2021 Aug 9;148(13):1648–64. doi: 10.1017/S0031182021001372 (PMC8564804; doi:10.1017/S0031182021001372)
Supplement: Supplementary file 1 [file S0031182021001372sup001.zip › S0031182021001372sup001/S0031182021001372sup003.docx]

**Online Resource Table S3** Comparative metrical data for cercariae of the “*Diplostomum baeri*” species complex

| **Species** | ***Diplostomum phoxini* (Faust, 1918)** | | | | ***D. baeri* (Dubois, 1937)** | ***Diplostomum* sp. Lineage 4 of Blasco-Costa *et al.* (2014)** | |
| --- | --- | --- | --- | --- | --- | --- | --- |
| **Snail host** | ***A. balthica*** | ***L. auricularia*** | ***L. peregra*** | ***L. peregra ovata*** | ***L. ovata*, *L. auricularia*** | ***R. peregra*** | ***R. peregra*** |
| **Source** | **Present study** | **Arvy and Buttner (1954)** | **Rees (1957)** | **Dönges (1969)** | **Niewiadomska and Kiseliene (1994)** | **Faltýnková *et al.* (2014)** | |
|  | **Range (Mean)** | **Mean** | **Mean** | **Range (Mean)^1^** | **Range (Mean)** | **Range (Mean)^2^** | **Range (Mean)^3^** |
| BL | 138–154 (143) | 125 | 125 | 151–178 (159) | 185–236 (216) | 156–265 (215) | 168–214 (189) |
| BW | 37–50 (41) | 35 | 41 | 45–60 (53) | 44–51 (50) | 52–82 (68) | 38–58 (48) |
| AOL | 52–59 (54) | 50 | – | 56–68 (62) | 40–57 (50) | 51–82 (62) | 41–61 (51) |
| AOW | 27–31 (28) | 20 | – | 22–30 (24) | 27–30 (29) | 34–44 (39) | 26–36 (32) |
| VSL | 30–34 (32) | 25 | 27 | 19–38 (28) | 20–27 (25) | 25–33 (29) | 22–31 (26) |
| VSW | 30–33 (32) | 25 | 27 | 19–38 (28) | 23–30 (29) | 26–37 (31) | 22–34 (28) |
| PHL | 11–14 | 12 | – | – | – | 10–16 (13) | 16–10 |
| PHW | 12–16 | 12 | – | – | – | 11–15 (13) |  |
| TSL | 212–226 (215) | 180 | 200 | 210–238 (224) | 244–281 (261) | 220–262 (243) | 234–280 (252) |
| TSW | 29–37 (33) | 20 | 24 | 34–43 (36) | 37 | 31–54 (44) | 34–48 (41) |
| FL | 212–239 (226) | 150 | 175 | 196–226 (210) | 251–273 (259) | 216–277 (241) | 200–259 (233) |
| AOW/VSW | 0.84–0.94 (0.90) | 0.80^d^ | – | (0.86)^4^ | (1.00)^4^ | 1.16–1.40 (1.26) | 1.00–1.30 (1.14) |
| TSL/BL | 1.38–1.69 (1.53) | 1.44^d^ | 1.60^d^ | (1.41)^4^ | (1.21)^4^ | 0.91–1.02 (0.96) | 1.20–1.43 (1.35) |
| TSL/FL | 0.95–1.03 (0.95) | 1.20^d^ | 1.14^d^ | (1.07)^4^ | (1.01)^4^ | 0.91–1.02 (0.96) | 0.98–1.19 (1.08) |

^1^ Heat-killed cercariae

^2^ Live cercariae

^3^ Fixed cercariae

^4^ Calculated from the mean values

*Note*: Host names as in the original articles
