# Supplementary material for: Molecular and morphological characterisation of Diplostomum phoxini (Faust, 1918) with a revised classification and an updated nomenclature of the species-level lineages of Diplostomum (Digenea: Diplostomidae) sequenced worldwide
Source: Parasitology. 2021 Aug 9;148(13):1648–64. doi: 10.1017/S0031182021001372 (PMC8564804; doi:10.1017/S0031182021001372)
Supplement: Supplementary file 1 [file S0031182021001372sup001.zip › S0031182021001372sup001/S0031182021001372sup004.docx]

**Online Resource Table S4.** Comparative metrical data for metacercariae of species of the *Diplostomum baeri* species complex

| **Species** | ***D. phoxini* (Faust, 1918)** | | | | ***Diplostomum* sp. Lineage 3 of Blasco-Costa *et al.* (2014)** | ***Diplostomum* sp. Lineage 4 of Blasco-Costa *et al.* (2014)** | ***Diplostomum* sp. Lineage 5 of Blasco-Costa *et al.* (2014)** |
| --- | --- | --- | --- | --- | --- | --- | --- |
| **Host** | ***P. phoxinus* (L.)** | | | | ***Salmo trutta fario* L.*, Salvelinus alpinus* (L.)** | ***Gasterosteus aculeatus* L.** | ***Salmo trutta fario* L., *Salvelinus alpinus* (L.)** |
| **Source** | **Present study** |  | **Rees (1955)** | **Lebedeva *et al.* (2021)** | **Faltýnková *et al.* (2014)** | **Faltýnková *et al.* (2014)** | **Faltýnková *et al.* (2014)** |
| **Feature** | **Range (Mean)^a^** |  | **Mean^a^** | **Range^b^** | **Range (Mean)^a^** | **Range (Mean)^a^** | **Range (Mean)^a^** |
| BL | 326–411 (358) |  | 344 | 215–325 | 447–601 (542) | 384–468 (417) | 363–501 (450) |
| BW | 145–227 (186) |  | 130 | 130–190 | 302–441 (376) | 210–309 (252) | 215–321 (283) |
| HL | 24–53 (42) |  | – | – | 30–68 (44) | 36–66 (49) | Not developed |
| PSL | 28–42 (34) |  | – | 25–40 | 89–118 (102) | 60–67 (64) | 49–66 (57) |
| PSW | 20–40 (30) |  | – | – | 32–49 (42) | 26–41 (34) | 28–35 (31) |
| OSL | 38–56 (49) |  | 46 | 35–50 | 51–67 (58) | 34–54 (46) | 36–45 (41) |
| OSW | 36–50 (44) |  | 41 | 25–50 | 46–66 (57) | 42–48 (46) | 40–61 (50) |
| PHL | 18–39 (31) |  | – | 20–40 | 29–55 (45) | 30–35 (32) | 30–37 (33) |
| PHW | 12–22 (18) |  | – | 10–20 | 20–40 (31) | 23–39 (29) | 29–40 (33) |
| VSL | 34–48 (43) |  | 43 | 30–40 | 41–68 (58) | 32–50 (44) | 45–61 (54) |
| VSW | 43–54 (47) |  | 50 | 30–50 | 68–72 (66) | 40–60 (49) | 51–64 (58) |
| HOL | 47–95 (71) |  | – | 45–70 | 128–195 (155) | 93–114 (103) | 92–146 (126) |
| HOW | 78–102 (91) |  | – | 50–80 | 162–238 (204) | 99–131 (112) | 164–188 (181) |
| No. of excretory concretions | 345–579 (454) |  | – | 678–796 | 450–600 | 400–450 | 450–500 |

^a^ Live metacercariae.

^b^ Fixed metacercariae.

*Abbreviations*: BL, body length; BW, body width; HL, hindbody length; PSL, pseudosucker length; PSW, pseudosucker width; OSL, oral sucker length; OSW, oral sucker width; PHL, pharynx length; PHW, pharynx width; VSL, ventral sucker length; VSW, ventral sucker width; HOL, holdfast organ length; HOW, holdfast organ width
