# Supplementary material for: Molecular and morphological characterisation of Diplostomum phoxini (Faust, 1918) with a revised classification and an updated nomenclature of the species-level lineages of Diplostomum (Digenea: Diplostomidae) sequenced worldwide
Source: Parasitology. 2021 Aug 9;148(13):1648–64. doi: 10.1017/S0031182021001372 (PMC8564804; doi:10.1017/S0031182021001372)
Supplement: Supplementary file 1 [file S0031182021001372sup001.zip › S0031182021001372sup001/S0031182021001372sup006.docx]

**Online Resource Table S1** Primers used for PCR amplification and sequencing

| **Marker** | **Primer name** | **Direction** | **Primer sequence (5'-3')** | **Usage** | **Cycling conditions** | **Reference** |
| --- | --- | --- | --- | --- | --- | --- |
| *Diplostomum phoxini* | | | | | | |
| *cox*1 | MplatCOX1dF | F | TGTAAAACGACGGCCAGTTTWCITTRGATCATAAG | PCR & Seq | 94 °C (3 min); 30× [94 °C (30 s); 50 °C (30 s); 72 °C (60 s)]; 72 °C (10 min) | Moszczynska *et al.* (2009) |
|  | MplatCOX1dR | R | CAGGAAACAGCTATGACTGAAAYAAYAIIGGATCICCACC | PCR & Seq |  |  |
|  | Plat-diploCOX1F | F | CGTTTRAATTATACGGATCC | PCR & Seq |  |  |
|  | Plat-diploCOX1R | R | AGCATAGTAATMGCAGCAGC | PCR & Seq |  |  |
| 28S rDNA | digl2 | F | AAGCATATCACTAAGCGG | PCR & Seq | 94 °C (3 min); 40× [94 °C (30 s); 55°C (30 s); 72°C (60 s)]; 72 °C (7 min) | Tkach *et al.* (1999) |
|  | 1500R | R | GCTATCCTGAGGGAAACTTCG | PCR & Seq |  | Tkach *et al.* (2003) |
|  | 900F | F | CCGTCTTGAAACACGGACCAAG | Sequencing |  | Olson *et al.* (2003) |
|  | 300 | R | GTTCATGGCACTCCCTTTCAAC | Sequencing |  | Lockyer *et al.* (2003) |
|  | ECD2 | R | CTTGGTCCGTGTTTCAAGACGGG | Sequencing |  | Littlewood *et al.* (2000) |
| ITS1-5.8S-ITS2 | D1 | F | AGGAATTCCTGGTAAGTGCAAG | PCR | 94 °C (3 min); 30× [94 °C (1 min); 56 °C (1 min); 72 °C (2 min)]; 72 °C (5 min) | Galazzo *et al.* (2002) |
|  | D2 | R | CGTTACTGAGGGAATCCTGGT | PCR |  |  |
|  | BD1 | F | GTCGTAACAAGGTTTCCGTA | Sequencing |  |  |
|  | BD2^b^ | R | TATGCTTAAATTCAGCGGGT | Sequencing |  |  |
| *Ampullaceana balthica* | | | | | | |
| ITS2 | RadITS2-News2F | F | TGTGTCGATGAAGAACGCAG | PCR & Seq | 94 °C (2 min); 30× [94 °C (30 s); 50 °C (30 s); 72 °C (30 s)]; 72 °C (7 min) | Almeyda-Artigas *et al.* (2000) |
|  | RadITS2-RixoR | R | TTCTATGCTTAAATTCAGGGG | PCR & Seq |  |  |

*Abbreviations*: *cox*1, cytochrome *c* oxidase subunit 1 gene; ITS, internal transcribed spacer; F, forward; R, reverse; Seq, sequencing

**References**

Almeyda-Artigas, R. J., Bargues, M. D., Mas-Coma, S., 2000. ITS-2 rDNA sequencing of *Gnathostoma* species (Nematoda) and elucidation of the species causing human gnathostomiasis in the Americas. J. Parasitol. 86, 537–544.

Galazzo D. E., Dayanandan S, Marcogliese DJ, McLaughlin JD. 2002. Molecular systematics of some North American species of *Diplostomum* (Digenea) based on rDNA-sequence data and comparisons with European congeners. Can. J. Zool. 80, 2207–2217.

Littlewood, D. T. J., Curini-Galletti, M., Herniou, E. A., 2000. The interrelationships of *Proseriata* (Platyhelminthes: Seriata) tested with molecules and morphology. Mol. Phylogenet. Evol. 16, 449–466.

Lockyer, A. E., Olson, P. D., Littlewood, D. T. J., 2003. Utility of complete large and small subunit rRNA genes in resolving the phylogeny of the Neodermata (Platyhelminthes): implications and a review of the cercomer theory. Biol. J. Linnean Soc. 78, 155–171.

Moszczynska, A., Locke, S. A., McLaughlin, J. D., Marcogliese, D. J., Crease, T. J., 2009. Development of primers for the mitochondrial cytochrome *c* oxidase I gene in digenetic trematodes (Platyhelminthes) illustrates the challenge of barcoding parasitic helminths. Mol. Ecol. Resour. 9, 75–82.

Olson, P. D., Cribb, T. H., Tkach, V. V., Bray, R. A., Littlewood, D. T. J., 2003. Phylogeny and classification of the Digenea (Platyhelminthes: Trematoda). Int. J. Parasitol. 33, 733–755.

Tkach V., Grabda-Kazubska B., Pawlowski J., Swiderski Z., 1999. Molecular and morphological evidences for close phylogenetic affinities of the genera *Macrodera*, *Leptophallus,* *Metaleptophallus*, and *Paralepoderma* (Digenea, Plagiorchioidea). Acta Parasitol. 44, 170−179.

Tkach, V. V., Littlewood, D. T. J., Olson, P. D., Kinsella, J. M., Swiderski, Z., 2003. Molecular phylogenetic analysis of the Microphalloidea Ward, 1901 (Trematoda: Digenea). Syst. Parasitol. 56, 1–15.
