# Supplementary material for: Molecular and morphological characterisation of Diplostomum phoxini (Faust, 1918) with a revised classification and an updated nomenclature of the species-level lineages of Diplostomum (Digenea: Diplostomidae) sequenced worldwide
Source: Parasitology. 2021 Aug 9;148(13):1648–64. doi: 10.1017/S0031182021001372 (PMC8564804; doi:10.1017/S0031182021001372)
Supplement: Supplementary file 1 [file S0031182021001372sup001.zip › S0031182021001372sup001/S0031182021001372sup007.docx]

**Online Resource Table S2** Prevalence of *Diplostomum phoxini* in *Ampullaceana* *balthica* collected during 2016, 2017 and 2019 in the River Ruhr at Neheim (Germany)

| **Site** | **May**  **(*n* = 255)** | **June**  **(*n* = 250)** | **July**  **(*n* = 266)** | **August**  **(*n* = 250)** | **September**  **(*n* = 264)** | **October**  **(*n* = 131)** | **November**  **(*n* = 104)** | **December**  **(*n* = 79)** |
| --- | --- | --- | --- | --- | --- | --- | --- | --- |
| B0 2016 | 0 (*n* = 20) | 0 (*n* = 21) | 0 (*n* = 2) | 0 (*n* = 31) | 0 (*n* = 28) | 0 (*n* = 20) | **4.5 (*n* = 22)** | 0 (*n* = 7) |
| B0 2017 | 0 (*n* = 28) | 0 (*n* = 23) | 0 (*n* = 27) | 0 (*n* = 21) | **10.0 (*n* = 20)** | 0 (*n* = 16) | – | – |
| B0 2019 | 0 (*n* = 30) | 0 (*n* = 30) | 0 (*n* = 30) | 0 (*n* = 30) | 0 (*n* = 30) | – | – | – |
| B1 2016 | 0 (*n* = 30) | 0 (*n* = 25) | 0 (*n* = 34) | 0 (*n* = 40) | **5.7 (*n* = 35)** | **8.7 (*n* = 23)** | 0 (*n* = 23) | 0 (*n* = 24) |
| B1 2017 | 0 (*n* = 33) | 0 (*n* = 40) | 0 (*n* = 41) | 0 (*n* = 31) | 0 (*n* = 43) | 0 (*n* = 31) | 0 (*n* = 35) | 0 (*n* = 31) |
| B1 2019 | 0 (*n* = 30) | 0 (*n* = 30) | **6.7 (*n* = 30)** | **3.3 (*n* = 30)** | **3.3 (*n* = 30)** | – | – | – |
| B2 2016 | 0 (*n* = 20) | 0 (*n* = 26) | 0 (*n* = 33) | 0 (*n* = 30) | 0 (*n* = 26) | 0 (*n* = 20) | 0 (*n* = 20) | 0 (*n* = 17) |
| B2 2017 | 0 (*n* = 40) | 0 (*n* = 25) | 0 (*n* = 39) | 0 (*n* = 7) | **13.6 (*n* = 22)** | 0 (*n* = 21) | 0 (*n* = 4) | – |
| B2 2019 | 0 (*n* = 24) | 0 (*n* = 30) | 0 (*n* = 30) | 0 (*n* = 30) | **3.3 (*n* = 30)** | – | – | – |
