# Supplementary material for: Molecular and morphological characterisation of Diplostomum phoxini (Faust, 1918) with a revised classification and an updated nomenclature of the species-level lineages of Diplostomum (Digenea: Diplostomidae) sequenced worldwide
Source: Parasitology. 2021 Aug 9;148(13):1648–64. doi: 10.1017/S0031182021001372 (PMC8564804; doi:10.1017/S0031182021001372)

Supplementary Figure S2 Phylogram from neighbour-joining (NJ) analysis of the *cox*1 sequence alignment (1,203 sequences; 407 nt) for 44 species/species-level lineages of *Diplostomum* (all sequences available on GenBank as of 29 June 2021). Outgroup: *Tylodelphys clavata*. Bootstrap support values ≥ 70 are provided above branches. The scale-bar indicates the expected number of substitutions per site. Sequences for isolates with updated species/lineage definitions are indicated in blue; sequences for re-identified/re-classified isolates are indicated in red (see Table 4 and Online Resource Table S5 for details).

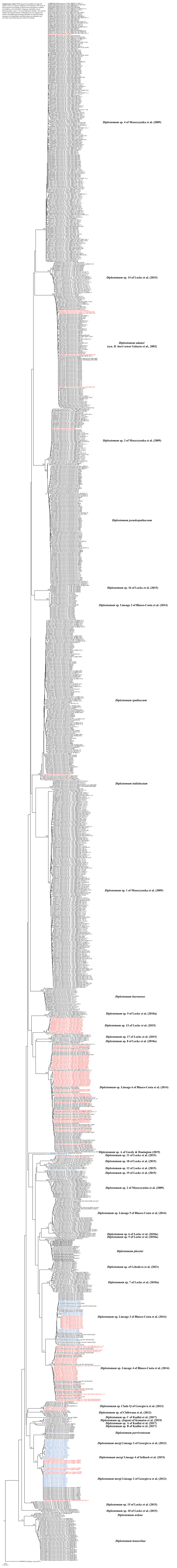

Supplement: Supplementary file 1 [file S0031182021001372sup002.pdf › S0031182021001372sup001/S0031182021001372sup002.pdf]
